# Sequence comparison, molecular modeling, and network analysis predict structural diversity in cysteine proteases from the Cape sundew, *Drosera capensis*

**DOI:** 10.1016/j.csbj.2016.05.003

**Published:** 2016-06-14

**Authors:** Carter T. Butts, Xuhong Zhang, John E. Kelly, Kyle W. Roskamp, Megha H. Unhelkar, J. Alfredo Freites, Seemal Tahir, Rachel W. Martin

**Affiliations:** a Department of Sociology, UC Irvine, USA; b Department of Statistics, UC Irvine, USA; c Department of Electrical Engineering and Computer Science, UC Irvine, USA; e Department of Chemistry, UC Irvine, USA; f Department of Molecular Biology & Biochemistry, UC Irvine, Irvine, CA, 92697 USA

**Keywords:** Cysteine protease, Carnivorous plant, Protein sequence analysis, Protein structure prediction, Protein structure network, Rosetta, Molecular dynamics, Digestive enzyme, *In silico* maturation

## Abstract

Carnivorous plants represent a so far underexploited reservoir of novel proteases with potentially useful activities. Here we investigate 44 cysteine proteases from the Cape sundew, *Drosera capensis*, predicted from genomic DNA sequences. *D. capensis* has a large number of cysteine protease genes; analysis of their sequences reveals homologs of known plant proteases, some of which are predicted to have novel properties. Many functionally significant sequence and structural features are observed, including targeting signals and occluding loops. Several of the proteases contain a new type of granulin domain. Although active site residues are conserved, the sequence identity of these proteases to known proteins is moderate to low; therefore, comparative modeling with all-atom refinement and subsequent atomistic MD-simulation is used to predict their 3D structures. The structure prediction data, as well as analysis of protein structure networks, suggest multifarious variations on the papain-like cysteine protease structural theme. This *in silico* methodology provides a general framework for investigating a large pool of sequences that are potentially useful for biotechnology applications, enabling informed choices about which proteins to investigate in the laboratory.

## Highlights

• 44 new cysteine proteases from the carnivorous plant *Drosera capensis* are described.
• Structure prediction and molecular dynamics simulation predict overall folds similar to papain.
• Functionally significant sequence and structural features are observed, including targeting signals and occluding loops.
• Several of the proteases contain a new type of granulin domain.
• Protein structure networks reveal global differences in interactions among chemical groups.

## Graphical abstract

**Figure:**
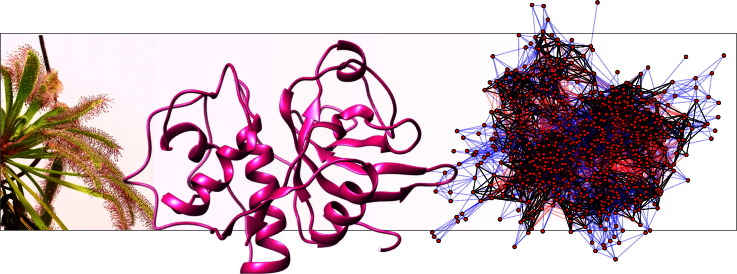

## 1 Introduction

The proteases of carnivorous plants present attractive targets for exploitation in chemical biology and biotechnology contexts. Carnivorous plants, such as *Drosera capensis*, whose prey capture functions take place in the open have been rigorously selected by evolution for the ability to digest large prey over long time periods, without assistance from physical disruption of prey tissue, and in competition with ubiquitous fungi and bacteria. These evolutionary constraints have selected for highly stable enzymes with a different profile of substate specificities and cleavage patterns from those found in animal digestive enzymes. Carnivorous plant digestive enzymes function at pH values ranging from 2–6, depending on the species [1], [2]. They also function over a wide range of temperatures; *Drosera* are endemic to every continent except Antarctica and both tropical and temperate species exist. In particular, the pH of *D. capensis* mucilage is around 5 [3], and temperatures in the Western Cape region of South Africa where these plants are found typically range from 5–30 °C.

Characterization of carnivorous plant digestive enzymes could lead to their use in a variety of laboratory and applications contexts, including analytical use in proteomics studies as well as preventing fouling on the surface of medical devices that cannot be treated under harsh conditions. New proteases may also prove useful for cleaving amyloid fibrils, such as those responsible for the transmission of prion diseases or the formation of biofilms by pathogenic bacteria. The characterization of aspartic proteases from the tropical pitcher plants (*Nepenthes* sp.) [4], [5], [6], has already led to useful advances in mass spectrometry-based proteomics applications, where the ability to digest proteins using a variety of cut sites is essential for identifying proteins and peptides from complex mixtures. Proteases from plant and animal sources are also important components of pharmaceutical preparations for gluten intolerance, arthritis, and pancreatic disease [7]. Characterizing proteases from carnivorous plants has the potential to diversify the toolbox of proteases with different functional properties that are available for these and other applications.

Plant cysteine proteases form a large and diverse family of proteins that perform cellular housekeeping tasks, fulfill defensive functions, and, in carnivorous plants, digest proteins from prey. It is typical for plants to contain many different cysteine protease isoforms; for instance, in the case of tobacco (*Nicotiana tabacum*), more than 60 cysteine protease genes have been identified [8]. Many of the cysteine proteases of interest are classified by the MEROPS database as family C1 [9], a broad class of enzymes including cathepsins and viral proteases as well as plant enzymes that function to deter herbivory. C1 proteases can operate as endopeptidases, dipeptidyl peptidases, and aminopeptidases [10]. In plants, many C1 enzymes are used to degrade proteins in the vacuole, playing many of the same roles as their lysosomal counterparts in animals [11]. They are also found in fruits, particularly unripe ones; this protease activity impedes insect feeding and also serves to cleave endogenous proteins during fruit ripening. Some families of cysteine proteases in plants have been subject to diversifying selection due to a molecular arms race between these plants and their pathogens; as plants produce proteases that suppress fungal growth, fungi evolve inhibitors specific to these proteases, driving the diversification of plant proteases involved in the immune response [12].

The plethora of paralogs found in a typical plant is indicative of the need for a range of different substrate specificities; this is particularly important in the case of carnivorous plants, which must digest prey proteins to their component amino acids. Aspartic proteases have long been implicated in *Nepenthes* pitcher plant digestion [4], [13], and more recently the cysteine protease dionain 1 has been confirmed as a major digestive enzyme in the Venus flytrap (*Dionaea muscipula*) [14]. In *D. capensis*, proteins from prey consititute the major nitrogen source for producing new plant tissue [15]. Given that plant carnivory appears to have evolved from defensive systems in general [16], and that the feeding responses are triggered by the same signaling pathway as is implicated in response to wounding [17], one would expect cysteine proteases to play a major role; here we investigate some of the many cysteine protease genes in *D. capensis* with the objective of adding to the portfolio of cleavage activities available for chemical biology applications. The *D. capensis* enzymes are particularly appealing for mass spectrometry-based proteomics applications, due to their ability to operate under relatively mild conditions, i.e. at room temperature and pH 5.

This study focuses on the C1 cysteine proteases from the Cape sundew (*D. capensis*), whose genome we have recently sequenced [18]. Here we use sequence analysis, comparative modeling with all-atom refinement and atomistic molecular dynamics (MD) simulation, and investigation of protein structure networks to identify structurally distinct subgroups of proteins for subsequent expression and biochemical characterization.

C1 cysteine proteases share a common papain-like fold, a property also predicted for the proteins studied here. Despite this conservation of the papain fold and critical active and structural residues, sequence analysis of the *D. capensis* cysteine proteases indicates that they represent a highly diverse group of proteins, some of which appear to be specific to the Droseraceae. In particular, a large cluster of proteases containing dionains 1 and 3 as well as many homologs from *D. capensis* has particular sequence features not seen in papain or other reference enzymes. Finally, a new class of granulin domain-containing cysteine proteases is identified, based on clustering of the granulin domains themselves.

Molecular modeling was performed in order to translate this sequence diversity into predicted structural diversity, which is more informative for guiding future experimental studies. Examination of the predicted enzyme structures potentially suggests diversity that may imply a variety of substrate preferences and cleavage patterns. The relationships between the shape of the substrate-recognition pockets and variation in substrate cleavage activity have been established for other plant cysteine proteases, including the ervatamins [19], the KDEL-tailed CysEP protease from the castor bean [20], and in dionain 1 [21]. The sequence-structure relationships outlined here suggest hypotheses that can be tested in the laboratory, providing a starting point for discovering novel enzymes for use in biotechnology applications. In most cases, the sequences have only weak identity to known plant proteases, making traditional homology modeling of dubious utility. Instead, we use Rosetta [22], [23] to perform comparative modeling with all-atom refinement, combining local homology modeling based on short fragments with de novo structure prediction. We then employ atomistic MD simulation of these initial structures in explicit solvent to produce equilibrated structures with corrected active site protonation states; these equilibrated structures serve as the starting point for further analysis.

Quality control was performed using both sequence alignment and inspection of the Rosetta structures; proteins that are missing one of the critical active residues (C 158 or H 292, papain numbering) were discarded, as were some lacking critical disulfide bonds or other structural features necessary for stability. After winnowing out sequences that are unlikely to produce active proteases, 44 potentially active proteases were chosen for further analysis. This methodology allows the development of hypotheses based on predicted 3D structure and activity, in contrast to focusing on the first discovered or most abundantly produced enzymes, enabling selection of the most promising targets for structural and biochemical characterization based on the priorities of technological utility rather than relative importance in the biological context.

## 2 Methods

### 2.1 Sequence Alignment and Prediction of Putative Protein Structures

Sequence alignments were performed using ClustalOmega [24], with settings for gap open penalty = 10.0 and gap extension penalty = 0.05, hydrophilic residues = GPSNDQERK, and the BLOSUM weight matrix. The presence and position of a signal sequence flagging the protein for secretion was predicted using the program SignalP 4.1 [25], while other localization sequences were identified using TargetP [26]. Structures were predicted using a three-stage process. First, an initial model was created for each complete sequence using the Robetta server [22]; the Robetta implementation of the Rosetta [23] system generates predictions from sequence information using a combination of comparative modeling and all-atom refinement based on a simplified forcefield. Second, any residues not present in each mature protein were removed, disulfide bonds were identified by homology to known homologs, and the protonation states of active site residues were fixed to their literature values. Finally, in the third phase, each corrected structure was equilibrated in explicit solvent under periodic boundary conditions in NAMD [27] using the CHARMM22 forcefield [28] with the CMAP correction [29] and the TIP3P model for water [30]; following minimization, each structure was simulated at 293 K for 500 ps, with the final conformation retained for subsequent analysis. This process was performed for the 44 protease sequences from *D. capensis*, as well as 10 reference sequences from other organisms (see below); where published structures were available, these were used as the initial starting model (following removal of heteroatoms and protonation using REDUCE [31] as required). For the 5 *D. capensis* sequences with granulin domains (as well as the two references with such a domain), steps (2) and (3) of the above were repeated after removal of the domain and any linking residues. This process provides predicted structures both with and without the domain in question. The PDB files corresponding to the equilibrated structures for all the proteins discussed in this manuscript are available in the Supplementary Information.

### 2.2 Network Modeling and Analysis

Each equilibrated protein structure was mapped to the network representation of Benson and Daggett [32] using custom scripts employing both VMD [33] and the statnet toolkit [34], [35] within the R statistical computing system [36]. Each vertex within the resulting protein structure network (PSN) represents a chemical group, with edges representing potential interaction as determined by proximity within the protein structure. PSNs were then compared using the structural distance technique of Butts and Carley [37], which provides a uniform way to compare the underlying structures of networks (i.e. graphs) with different vertex sets; this involves mapping both graphs onto a common vertex set (adding isolated vertices to the smaller graph as needed) such that the differences between the two mapped networks are minimized with respect to an underlying metric. The value of this metric after mapping is the structural distance. Here, distances were computed between unlabeled graphs based on an underlying Hamming metric, and can be interpreted as the minimum number of edge changes required to transform a member of the isomorphism class of the first graph (i.e., the set of all graphs having the same underlying typology) into a member of the isomorphism class of the second (or vice versa). The raw structural distance between each pair of PSNs was then normalized by graph order, yielding a metric corresponding to edge changes per vertex. Normalized structural distances between PSNs were analyzed via metric multidimensional scaling and hierarchical clustering using R. Additional network visualization and analysis was performed using the sna library [38] within statnet.

## 3 Results and Discussion

### 3.1 *D. capensis* Cysteine Proteases Cluster Into Distinct Families Based on Resemblance to Known Homologs

**Fig. 1:**
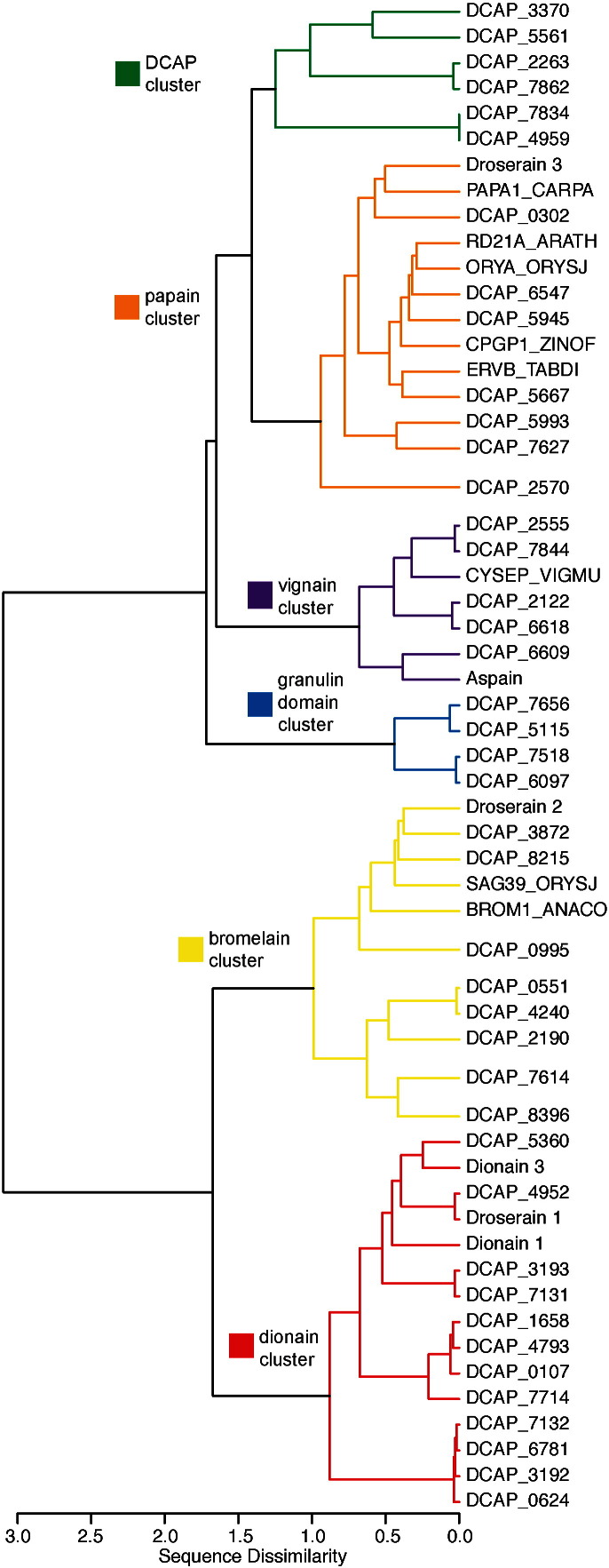
Clustering of cysteine protease sequences identified from the *D. capensis* genome. Many are homologous to known plant cysteine proteases, including dionain 1 and dionain 3 from the Venus flytrap, *D. muscipula*. Dissimilarity between clusters is defined by the *e*-distance metric of Székely and Rizzo [45] (with *α* = 1), which is a weighted function of within-cluster similarities and between-cluster differences with respect to a user-specified reference metric. The underlying input metric employed here is the raw sequence dissmilarity (1 − (%identity)/100).

All *D. capensis* sequences previously annotated as coding for MEROPS C1 cysteine proteases using the MAKER-P (v2.31.8) pipeline [39] and a BLAST search against SwissProt (downloaded 8/30/15) and InterProScan [40] were clustered by sequence similarity. Several previously-characterized cysteine proteases that have been identified from other plants are also included as reference sequences. Clustering of the *D. capensis* cysteine protease sequences reveals a broad range of cysteine protease types, some of which are homologous to known plant proteases (Fig. 1). Three of the six clusters contain only proteins from *D. capensis* or the related Venus flytrap *D. muscipula*, while many of the reference sequences cluster together despite coming from a variety of different plant species from diverse orders including both monocots and eudicots (Supplementary Table S1). The general types of plant protease features found correlate well with previous surveys of cysteine proteases in *Arabidopsis thaliana*[41], *Populus* sp. [42], and more recently, soybeans [43] and a broader group of plant proteases from a variety of species [44].

### 3.2 Residues Conserved in *D. capensis* Cysteine Proteases Include Active Sites and Important Sequence Features

A defining feature of C1A cysteine proteases is the Cys-His catalytic dyad, which is often accompanied by an Asn residue that stabilizes the protonated catalytic His [46], [47]. The mechanism of these enzymes requires using the thiolate group on the deprotonated cysteine as a nucleophile to attack a carbonyl carbon in the backbone of the substrate. Preliminary sequence alignments comparing putative cysteine proteases from *D. capensis* were used to discard sequences lacking the conserved Cys and His residues of the catalytic dyad due to either substitution or truncation. Other conserved features were observed in many of the sequences, but were not treated as necessarily essential for activity. Reference sequences used include zingipain 1 from *Zingiber officianale* (UniProt P82473), pineapple fruit bromelain (*Ananas comosus*, UniProt O23791), RD21 from *A. thaliana* (UniProt P43297), oryzain alpha chain (UniProt P25776) and SAG39 (UniProt Q7XWK5) from *Oryza sativa* subsp. *japonica*, ervatamin b from *Tabernaemontana divaricata* (UniProt P60994), and dionains 1 and 3 from the related *D. muscipula* (UniProt A0A0E3GLN3, and A0A0E3M338, respectively). Several of the reference sequences, e.g. zingipain-1 [48], were characterized by mass spectrometric analysis of the mature enzyme; these sequences therefore lack the signal peptide and pro-sequence found in the initially transcribed sequence (see below).

Sequence alignments for the individual clusters (Supplementary Figs. S1–S7) are annotated to highlight individual amino acid properties, residues conserved within the cluster and/or shared with papain, as well as functional sequence features, as described in detail in the S.I. In addition to the cluster-specific reference sequences, all clusters include papain (*Carica papaya*, UniProt P00784) in order to have a common reference for all the C1A proteases discussed in this work.

Most of the clusters are named after a reference sequence or a distinguishing feature of its members. The DCAP cluster is highly diverse, yet it contains only sequences from *D. capensis*. The papain cluster contains many of the reference sequences, as well as several *D. capensis* proteases, some of which have granulin domains (Figs. S2 and S3), a feature that is peculiar to plant cysteine proteases. The vignain cluster (Fig. S4) contains vignain from *Vigna mungo* (UniProt P12412) as well as *D. capensis* homologs. Many of the proteins in the vignain cluster have C-terminal KDEL tags, indicating retention in the ER lumen, suggesting that they are involved in germination and/or senescence. In the granulin domain cluster (Fig. S5), every sequence but one contains a granulin domain connected to the catalytic domain by a proline-rich linker of about 40 residues; the one exception is truncated after the proline-rich region. Several sequences in the papain cluster also contain granulin domains, however the Pro-rich linkers in those sequences contain only about 16 residues and the sequence identity between the two types of granulin domains themselves is not high. The bromelain cluster (Fig. S6) contains homologs of both defensive and senescence-related enzymes. Every sequence in the dionain cluster (Fig. S7) contains an extra Cys residue immediately prior to the active site Cys. This CCWAF structural motif has been previously observed in the Arabidobsis protein SAG12 and homologs [44]; however, the function of the double Cys in unknown. It may have cataytic relevance, perhaps providing a second nucleophilic thiolate or operating as a redox switch.

Like many other proteases, the papain-family enzymes are expressed with an N-terminal pro-sequence blocking the active site. This sequence is cleaved during enzyme maturation, often upon the protein's entering a low-pH environment. This pro-sequence was found in most of the C1A proteases from *D. capensis* (highlighted with pink boxes in Figs. S1–S7 in the SI). Plant C1A protease pro-sequences are often bioactive in their own right, acting as inhibitors of exogenous cysteine proteases. This enables them to deter herbivory by insects [49], nematodes [50], and spider mites [51], protecting the plants from damage. This can be technologically exploited by producing transgenic crop varieties with protective cysteine proteases they would otherwise lack [52]. This approach has proven useful in protecting crops from Bt-resistant pests [53]. Despite some variation in the lengths of the C-terminal and N-terminal regions, all the cysteine proteases investigated here show substantial similarity in the pro-sequences; in particular, the ERFNIN motif (EX _3_RX _3_FX _2_NX _3_IX _3_N) often found in the pro-sequence of C1A proteases [54] is conserved in many sequences spanning all the clusters. Interestingly, the alternative sequence EX _3_RX _3_FX _2_NX _3_AX _3_Q, which is characteristic of the RD19 family of plant cysteine proteases, is found in only one of the *D. capensis* proteases, DCAP_3370 in the DCAP cluster. For all previously uncharacterized sequences, SignalP 4.1 [25] was used to predict the location of the signal sequences, if any, while the pro-sequences were predicted by sequence similarity and structural homology to papain. These sequence annotations were then used as the basis for further structure prediction and functional analysis.

In addition to the common sequence features in the N-terminal pro-region, other variations are observed, such as the presence of C-terminal granulin domains in some sequences and extra insertions that may be responsible for specific activities in others. Examples of organelle-specific targeting sequences are observed; several sequences have a C-terminal KDEL sequence targeting them for retention in the endoplasmic reticulum, while others have targeting sequences indicating their destination in the cells, including signals indicating transport to the vacuole (NPIR, but not FAEAI or LVAE) or the peroxisome (SSM at the C-terminus). The level of sequence conservation among the members of each cluster varies dramatically, as can be seen in Fig. S8, where sequence conservation is mapped onto the structure of a representative member of each cluster. The sequences in the DCAP cluster are less closely related to each other than the members of any of the other clusters, and some are homologous to reference sequences used by Richau et al. [44].

DCAP_2263 and DCAP_7862 belong to the Richau aleurain (cathepsin H) cluster. In humans, cathepsin H is an aminopeptidase that processes neuropeptides in the brain [55], as well as acting as a lysozomal protein in other tissues. Its barley (*Hordeum vulgare*) homolog, aleurain, has both aminopeptidase and endopeptidase activity [56], suggesting that DCAP_2263 and DCAP_7862 may have both types of activity as well. This hypothesis is supported by the presence of the Cathepsin H minichain sequence in its plant orthologs, as discussed in the section devoted to these proteins. DCAP_3370 is related to the Richau RD19 (cathepsin F) cluster, and is the only protease in this set that contains the characteristic pro-sequence motif (EX _3_RX _3_FX _2_NX _3_AX _3_Q), of the RD19 (cathepsin F) family. Human cathepsin F is distinguished by its unusually long pro-domain, which is approximately 100 residues longer than that of other cysteine proteases and adopts a cystatin fold [57]. In contrast, the pro-sequence of DCAP_3370 is about 140 residues, typical for a plant cysteine protease. The last enzyme in the DCAP cluster, DCAP_5561 is not closely related to anything in either reference set. A BLAST search yields numerous matches to uncharacterized predicted cysteine proteases from a variety of plant genomes, however, the specific function of this enzyme remains enigmatic.

### 3.3 Molecular Modeling Predicts Many Variations on the Papain Structural Theme

Carnivorous plants require a variety of proteases with different substrate affinities and cleavage sites to effectively digest the proteins from their prey, in addition to the standard spectrum of protease activities required by all plants. Cysteine protease activity has previously been inferred from biochemical activity assays of the digestive fluids of *D. indica*[58], and dionain 1 from *D. muscipula* has been structurally and biochemically characterized [21]. However, with the exception of the nepethesins and dionain 1, these enzymes have yet to be extensively investigated. In this study, 44 cysteine proteases with moderate sequence homology to papain-like enzymes of known structure have been identified from the genome of *D. capensis*. For each putative functional protease, the structure of the full-length sequence, including the signal peptide, the pro-domain(s), and the granulin domain if present, was predicted using Rosetta. The resulting sequence was then subjected to *in silico* maturation, where known features of these enzymes were corrected, including addition of disulfide bonds and removal of pro-sequences and granulin domains, followed by equilibration using MD.

**Fig. 2:**
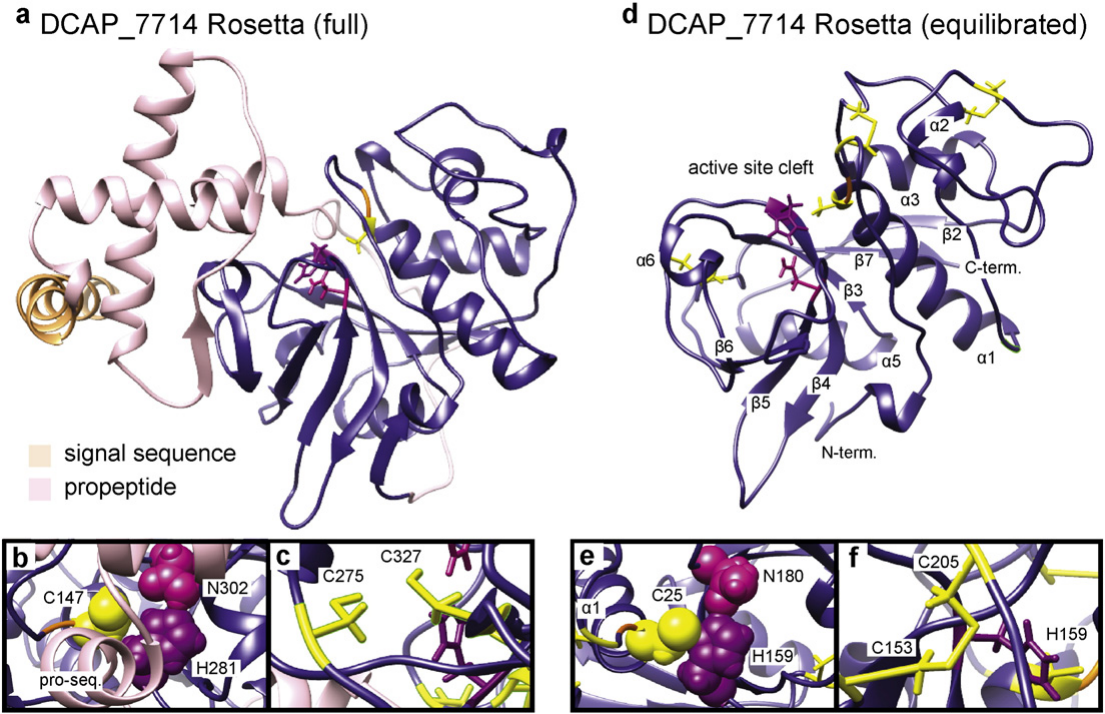
Predicted structures of DCAP_7714 before (a) and after (d) *in silico* maturation. (b) The active site residues (shown as space-filling models and with zymogen numbering) are in an unfavorable conformation prior to adjustment of their protonation states and equilibration in explicit solvent, whereas after equilibration. (e) the confornation is more consistent with that of an active cysteine protease (the same active site residues are shown but with mature sequence numbering). (c) In the initial Rosetta structure, the Cys rotamers (shown here for residues C275 and C327, zymogen numbering) are not generally in the ideal conformation for disulfide bonding, even in cases where it is expected. (f) Disulfide bonds (positions determined using sequence homology to papain) were added before equilibration. The residues shown here are the same as in panel (c), but with mature sequence numbering .

The *in silico* maturation and equilibration process allows for refinement of the initial Rosetta structure predictions. The Rosetta structure for a representative full-length protease (DCAP_7714) is shown in Fig. 2a. The full-length sequence consists of the active region, a secretion signal peptide (light orange), and an N-terminal pro-sequence (pink). The core sequence making up the mature form of this enzyme (dark blue) is structurally similar to papain, with two domains of approximately equal size, one primarily *α*-helical and the other mostly composed of *β*-strands, with the active site cleft between them. The inset in Fig. 2b shows the active Cys (yellow)/His (purple) dyad as well as the stabilizing Asn residue (magenta). In general, the structures predicted by Rosetta provide reasonable estimates for the overall folds of these enzymes, given their homology to papain. However, some details such as side chain rotamers are not perfectly consistent with known structures of papain-like enzymes. In particular, in the Rosetta structure, the S of the active cysteine is rotated up and away from the active histidine, and the side chains of cysteine residues predicted to be involved in disulfide bonds are not in the correct orientations (Fig. 2c). In order to generate more realistic structures for network analysis, *in silico* maturation and equilibration were performed. The equilibrated structure of the mature form of DCAP_7714 is shown in Fig. 2d. Secondary structure elements are numbered according to the structure of Than et al. for the homologous *Ricinus communus* CysEP enzyme [20]. The protonation states of the active Cys and His were modified to reflect their expected states in the mature enzyme, resulting in more realistic side chain conformations in the equilibrated structure (Fig. 2e). The disulfide bonds were added to the structure before equilibration (Fig. 2d) based on homology to papain (and RD21A_ARATH in the case of enzymes containing a granulin domain).

**Fig. 3:**
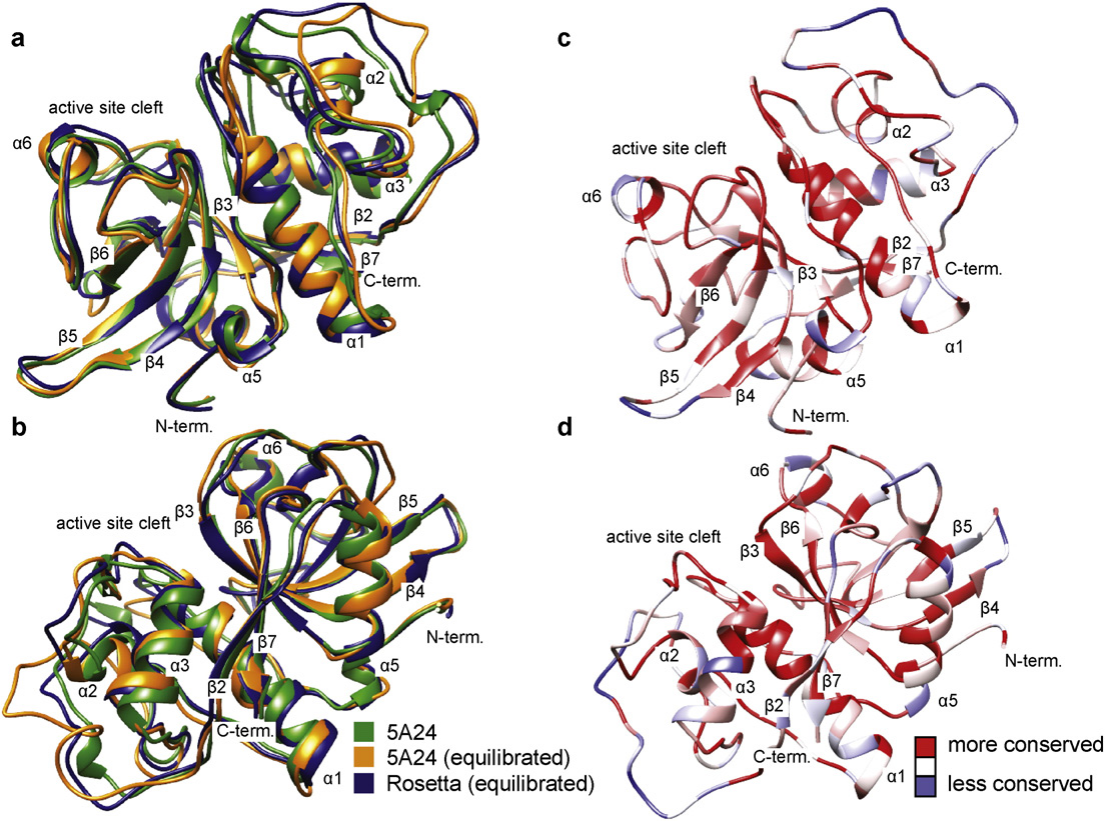
a. and b. Structural comparison of the X-ray crystal structure of dionain 1 (PDBID 5A24) [21] (green) with the same structure after equilibration in solvent (orange) and the structure predicted by Rosetta after equilibration (blue), two different views. The Rosetta structure predicts all important secondary structure features observed in the crystal structure. Equilibration of the crystal structure in solvent prior to docking studies results in conformational changes to flexible loops as well as repositioning of side chains. c. and d. The percent conservation for each residue in the consensus sequence of the entire dionain cluster is plotted on the structure of dionain 1. Highly conserved residues tend to cluster in sequence regions where the predicted structure coincides with the observed structure, consistent with the idea that structurally important residues are strongly conserved.

As a validity check on our approach, we provide a comparison between our structural predictions (initial and refined) and an out-of-sample observation. The recently solved x-ray crystal structure of dionain 1 was published after our initial prediction and equilibration of this protein was performed, and hence this structure could not have been in the Rosetta training set. In Fig. 3a and b, the crystal structure (5A24, green) [21] is shown overlaid with its MD equilibrated counterpart (orange), and the structure predicted by Rosetta, after *in silico* maturation (blue). The Rosetta structure shows excellent agreement with the experimentally determined crystal structure, with nearly complete overlap of the major secondary structure elements, e.g. helices 1, 3, and 5, as well as the *β*-sheet formed by *β*-strands 3, 4, 5, and 6. As expected, substantially less agreement is observed in the loop regions, such as the flexible linkers between helices 1 and 2 and between helix 3 and strand 4. Molecular dynamics equilibration of the crystal structure in TIP3P solvent also results in movement of the loop regions, as is evident from comparison of the green and orange structures. Examination of the sequence conservation map of the dionain cluster plotted on the dionain 1 structure (Fig. 3 c and d) reveals that the most strongly conserved residues coincide with sequence regions predicted well by Rosetta, e.g. helix 1 and strands 3, 4, 5, and 6. In contrast helix 2, the loop regions, and the N-terminus have a higher RMSD between the crystal structure and the predicted structure, and display lower sequence conservation. This is consistent with the hypothesis that one reason for conservation of particular residues is that they are important for maintaining the structure. Overall, the close match between the predicted and observed dionain 1 structures indicates that our approach can provide excellent structural predictions within this class of proteins.

### 3.4 Some Cysteine Proteases Are Targeted to Specific Locations

**Fig. 4:**
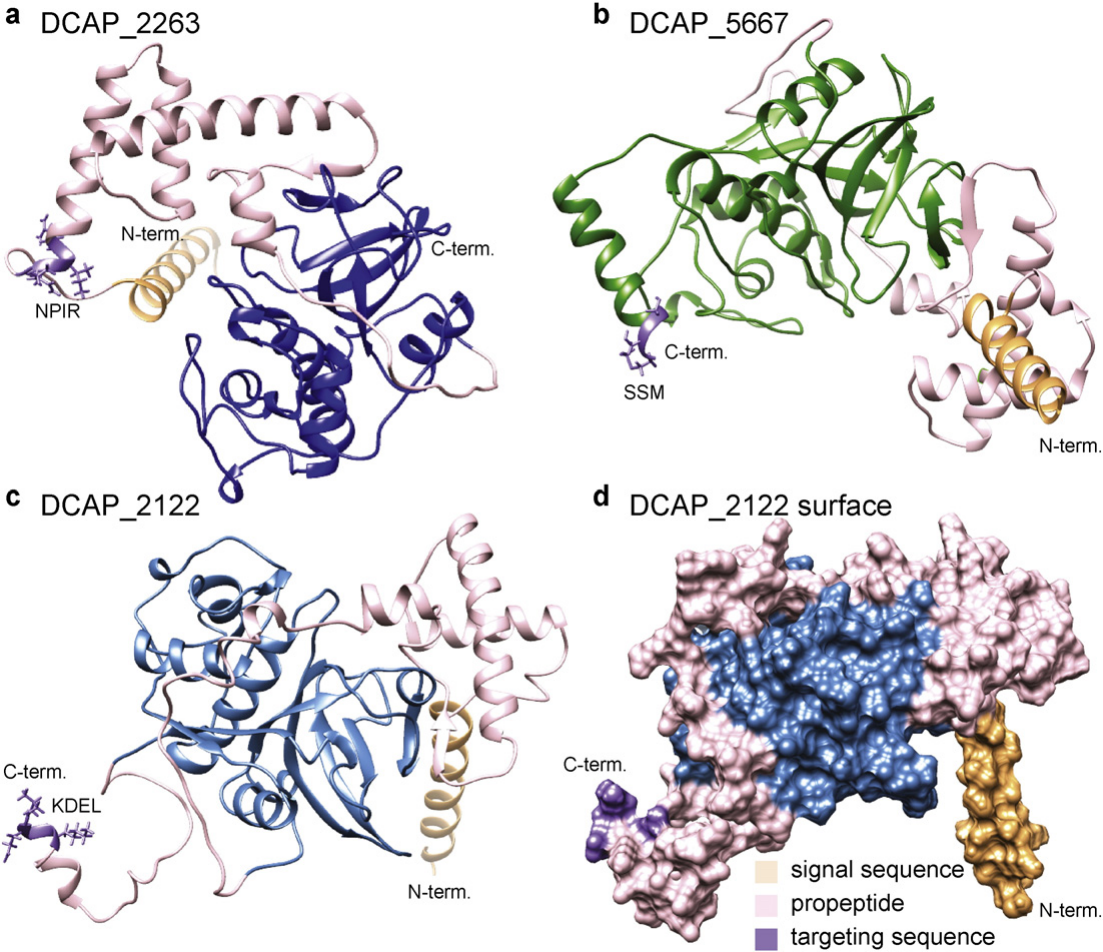
Predicted structures for three full-length cysteine proteases. The secretion signals are highlighted in light orange, the pro-sequences in pink, and the localization tags in light purple. a. DCAP_2263 contains the target sequence NPIR, indicating localization to the vacuole. b. DCAP_5667 ends in the tripeptide SSM at the extreme C-terminus, indicating transport to the peroxisome c. and d. DCAP_2122 ribbon diagram and surface model, respectively. DCAP_2122 ends in the ER-retention signal KDEL, indicating that it is retained in the ER lumen.

Several of the cysteine proteases identified from *D. capensis* contain known targeting signals that mark the protein for delivery to specific cellular locations. The most common such signal is the N-terminal signal peptide targeting the protein for secretion. As expected, the majority of proteins in this set contain such a secretion signal. In plants, the secretory pathway delivers proteins to the vacuole, the vacuolar membrane, the cell wall, and the plasma membrane. In *D. capensis*, digestive enzymes are also expected to be secreted into the mucilage. In addition to the N-terminal signal sequences, tri- or tetrapeptides indicating that the protein is destined for a particular subcellular compartment are also found in many cases. Fig. 4 shows the structures predicted by Rosetta for three full-length cysteine proteases containing targeting signals, DCAP_2263, DCAP_5667, and DCAP_2122. Ribbon diagrams are shown for all three enzymes; a surface is also shown for DCAP_2122 in order to assist with visualization of the relationship of the pro-sequence, N-terminal signal peptide, and C-terminal localization sequence to the rest of the protein. The positioning of the pro-sequences (pink) and signal peptides (light orange) is highly variable, although in each example the pro-sequence blocks the active site and the signal sequences and other localization tags (light purple) are in highly exposed positions as expected based on their function.

In plants, the subsequence NPIR in the N-terminal region of a protein indicates targeting to the vacuole, a large acidic compartment that is specific to plant cells and serves the same function as the lysozome in animal cells. These compartments, which often occupy most of the volume of the cell, contain a variety of hydrolases, including both aspartic and cysteine proteases, which normally act to recycle damaged or unneeded cellular components. Upon infection by viruses or fungal pathogens, the vacuole can also fuse with the plasma membrane to release defensive proteases into the extracellular space. Two putative vacuolar proteases, (DCAP_2263 and DCAP_7862) are found in the DCAP cluster. The NPIR tag is located in an exposed position between the secretion signal and the beginning of the N-terminal pro-sequence, as shown for DCAP_7862 in Fig. 1a. These proteases display sequence homology to mammalian cathepsin H, a lysozomal protein that is important in development and also implicated in cancer proliferation [59], [60].

**Fig. 5:**
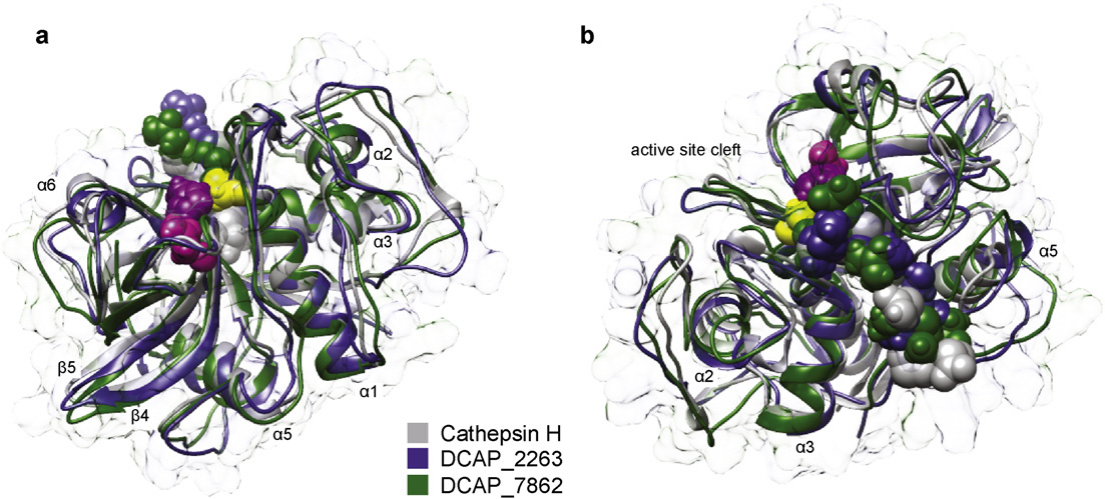
Predicted structures for two vacuolar cysteine proteases (DCAP_2263, blue and DCAP_7862, green) with sequence homology to cathepsin H (PDBID: 8PCH gray). The active site residues and the minichain are shown as space-filling models. a. One side of the active site cleft is open and accessible to substrate. b. The other side of the active site cleft is blocked by the minichain. In cathepsin H, this partial occlusion of the active site confers aminopeptidase specificity.

In human cathepsin H, aminopeptidase activity is modulated by the minichain sequence (EPQNCSAT). DCAP_2263 and DCAP_7862 (and aleurain, but no others in this set) contain the sequence AAQNCSAT, which may have a similar function. The hypothesis that this plant-specific minichain serves a similar role in modulating the substrate specificity is supported by comparing the predicted structures with the crystal structure of porcine cathepsin H (PDBID: 8PCH) [61]. Fig. 5 shows the predicted structures of mature DCAP_2263 (blue) and DCAP_7862 (green) overlaid with the crystal structure of porcine cathepsin H (gray). The predicted structures of the plant proteins coincide with the porcine protein in the major secondary structure elements, albeit with substantial variation in loops and linkers. The minichain sequence (EPQNCSAT in the porcine protein and AAQNCSAT in the *D. capensis* proteins) occupies a similar position in all three structures, allowing substrate approach to the active site cleft from one side (Fig. 5a), but not the other (Fig. 5b). Biochemical characterization of human cathepsin H has shown that deletion of the minichain abolishes aminopeptidase activity [62], making this protein a standard endopeptidase. Based on sequence homology and examination of the predicted structures, we hypothesize that this sequence plays a similar role in modulating the substrate specificity and activity patterns of DCAP_2263 and DCAP_7862.

Other proteases are targeted to the peroxisomes, organelles that bud from the ER membrane and primarily break down long-chain fatty acids, but are also involved in the synthesis of functional small molecules, such as isoprenoids, polyamines, and benzoic acid [63]. Some proteases in the peroxisome are involved in the maturation of other enzymes imported to this organelle, as well as disposal of oxidized proteins that build up in this challenging redox environment [64]. Others are active during different developmental stages, such as differentiation of seed glyoxysomes to mature leaf peroxisomes [65]. The most common type of targeting signal for transport to the peroxisome is one of several C-terminal tripeptides. The canonical example is SKL, but others have been discovered in a variety of plant proteins [66]. DCAP_5667, which is in the papain cluster (Fig. S3), has the tripeptide SSM at its extreme C-terminal end, indicating targeting to the peroxisome. DCAP_7656, which is in the granulin domain cluster (Fig. S5), contains the SKL sequence not at the C-terminal end, but at a highly exposed position near the C-terminus, suggesting possible peroxisome targeting for this protein also. DCAP_7656 contains the proline-rich linker common to this cluster, but its granulin domain is truncated. Another possibility is that the short sequence region following the SKL tripeptide may be cleaved under some circumstances, acting as a switch that determines whether this enzyme is sent to the peroxisome or elsewhere. Peroxisome-targeted proteases represent attractive targets for biotechnological studies, because they are optimized to remain stable and maintain their activity under harshly oxidizing conditions.

Proteins with the sequence KDEL at the C-terminus are retained in the lumen of the endoplasmic reticulum, enabling them to be stored in specialized vesicles as zymogens and released to mediate programmed cell death in response to a stressor or during a particular developmental phase. KDEL-tailed proteases such as vignain from *V. mungo* and CysEP from *R. communis* play an important role during germination, when proteins stored in endosperm tissue are degraded for use as the cotelydons develop. A C-terminal pro-peptide including the KDEL tag is removed along with the N-terminal pro-sequence during maturation, to yield the soluble, active enzyme [67]. The crystal structure and biochemical characterization of a homologous KDEL-tailed protein from the castor bean indicates that this enzyme has a strong preference for large, neutral amino acids in the substrate peptides, and has an unusually large and possibly flexible substrate-binding pocket that can accommodate a variety of sidechains, including proline [20].

### 3.5 Several Discovered Proteases Possess Novel Granulin Domains

Cysteine proteases with a C-terminal granulin domain are specific to plants, where they are involved in response to dessication or infection by pathogenic fungi [68]. This type of domain is found in two of the *D. capensis* protease clusters, the papain cluster Fig. S3) and the granulin domain cluster (Fig. S5). The reference sequences RD21A (RD21A_ARATH) from arabidopsis and oryzain (ORYA_ORYSJ) from rice both contain granulin domains, as do three proteins in the papain cluster (Fig. S3) and three in the granulin domain cluster (Fig. S5). An additional two sequences in the papain cluster and one in the granulin domain cluster contain truncated versions that do not contain all four cysteine residues necessary to form the two disulfide bonds stabilizing the granulin domains. The granulin domain is separated from the catalytic domain by a proline-rich linker region. In RD21A, which is found in both the vacuole and the ER bodies [69], the granulin domain is removed from the mature enzyme. Maturation within the vacuole is relatively slow and involves accumulation of an intermediate where the N-terminal pro-sequence is removed and the C-terminal granulin domain remains attached [70]. This intermediate species forms aggregates that slowly release active enzyme following cleavage of the granulin domain, which is performed by RD21 itself [71]. This suggests that aggregation mediated by the granulin domain provides a mechanism for regulating protease activity during leaf senescence.

**Fig. 6:**
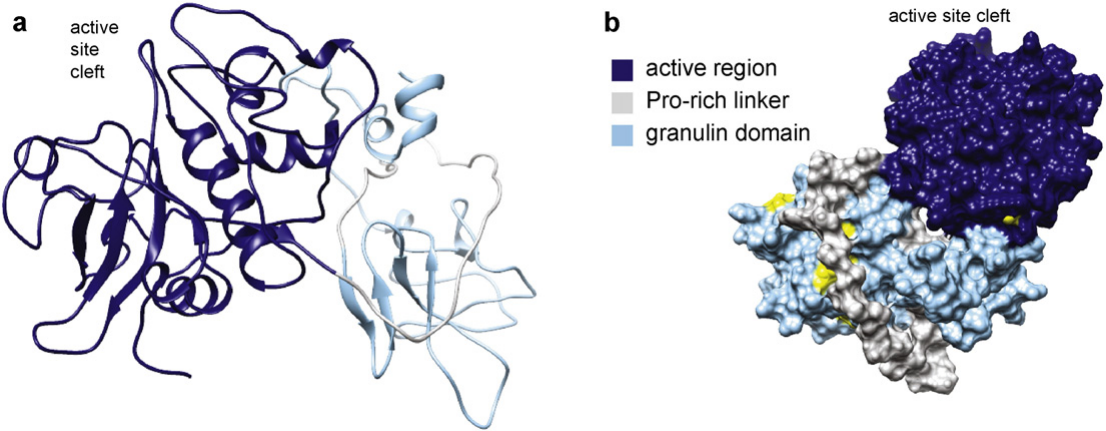
a. Ribbon diagram for the predicted structure for a representative member of the granulin domain cluster (DCAP_5115), showing the catalytic domain (dark blue), the proline-rich linker (gray) and the granulin domain (light blue). b. Surface representation of the same structure rotated to show how the proline-rich linker interacts with the granulin domain.

**Fig. 7:**
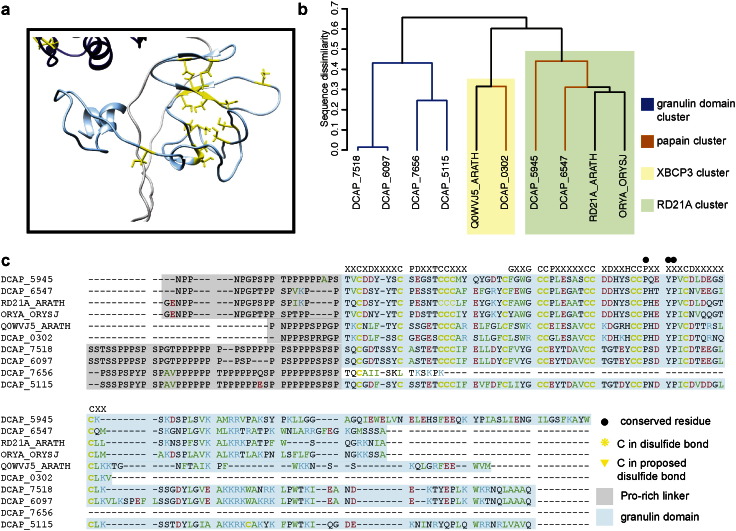
a. Ribbon diagram of the DCAP_5115 granulin domain, with cysteine residues highlighted in yellow. b. Cluster analysis of granulin domains from *D. capensis* cysteine proteases and reference sequences. Solid colors denote membership in the clusters of Fig. 1, while the transparent boxes correspond to the clusters previously identified by Richau et al. [44]. Notably, the *D. capensis* granulin domain cluster appears to represent a new type of plant cysteine protease granulin domain. c. Sequence alignment of all the granulin domains found in the *D. capensis* cysteine proteases with reference sequences.

The granulin domain is attached to the catalytic domain by a proline-rich linker of variable length, as illustrated in Fig. 6. Granulins in animals act as growth factors, and contain distinct sequence and structural features: the characteristic sequence motif consists of four pairs of cysteine residues, with single conserved cysteines on both sides, and the resulting fold consists of *β* hairpins held together by disulfide bonds [72]. In plants, the granulin domain has two additional cysteines and an insertion of 6 residues between the first two Cys pairs, slightly modifying the structure (Fig. 7a). Clustering of the granulin domains themselves, separately from the catalytic domains, yields three clusters (Fig. 7b), two of which contain proteins from the *D. capensis* papain cluster and one of which is made up entirely of proteins from the *D. capensis* granulin domain cluster. The cluster analysis of Richau et al. [44] identified two subfamilies of granulin domain-containing cysteine proteases; comparison with those results places DCAP_0302 in their XBCP3 cluster, while DCAP_5945 and DCAP_6547 are in their RD21A cluster. Notably, the *D. capensis* granulin domain cluster represents a new subfamily of plant cysteine proteases that is not closely related to either of the previously described subfamilies.

The key sequence region of the canonical animal granulin motif is shown above the sequence alignment for comparison (Fig. 7c). The plant granulin sequences have two distinguishing features; an additional conserved Cys residue is present immediately after the first conserved CC pair in the animal sequence, and a 6-residue insertion containing another conserved C is present between the first and second CC pairs. In the granulin domain cluster, there is also a one-residue deletion between the first two conserved Cys residues. The first conserved glycine in the animal sequence is not conserved in the plant granulin domains, and in fact all of the examples shown here contain a bulky residue (F, Y, or L) at that position.

### 3.6 Protein Structure Networks Reveal a Tripartite Pattern of Structural Differentiation

In addition to the presence or absence of specific features, identifying broader patterns of structural differentiation can be helpful when selecting putative proteins for expression and characterization: proteins within different structural subgroups may differ with respect to other biophysically important properties such as thermal stability, substrate affinity pattern, overall activity, or aggregation propensity, and choosing a structurally diverse sample thus has the potential to maximize the chance of identifying proteins with functionally significant variation. Protein structure networks (PSNs) are a useful tool for such exploration, as they directly represent patterns of potential interaction among chemical groups rather than e.g. side chain dihedral angles or other properties that may vary substantially without inducing significant changes in protein function. Here, we employ the PSN representation of Benson and Daggett [32], which associates a vertex with each functionally distinct chemical group in the protein and assigns edges between vertices on the basis of their potential for direct interaction (as determined by a combination of inter-atomic distances and the identity of the groups in question). Using the structural distance approach of Butts and Carley [37], we can then directly compare protein structures via their PSNs.

**Fig. 8:**
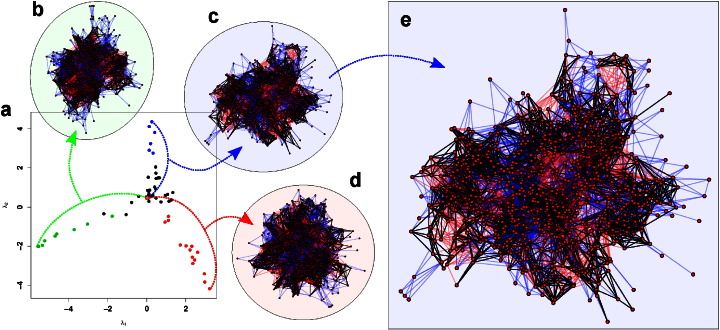
a. Two-dimensional metric MDS solution for cysteine protease PSNs, based on structural distance; Euclidean distances between points approximate the structural distances between their corresponding PSNs. Protease PSNs show three distinct patterns of continuous variation from a central group of structures (black dots); the two-dimensional MDS solution is corroborated by the results of a four-group hierarchical clustering solution (indicated by point color), which also finds one central and three elongated peripheral groups. b., c., d., show minimum-distance mappings between the most central PSN (i.e., the PSN with the smallest median structural distance to all other PSNs, shown in orange) and the most extreme PSN on each axis of variation (see dotted lines). Black edges in each mapping show edges present in both PSNs, blue edges indicate edges present in the center but not the extreme PSN, and red edges indicate edges present in the extreme PSN but not the center. Differences between central and extreme PSNs are not localized to any particular location, but broadly diffused throughout the each graph. e. Detail of extreme/central PSN mapping for the blue axis, showing concentrated regions of edge addition (blue) and subtraction (red) as one approaches the center.

Fig. 8a shows a metric multidimensional scaling (MDS) representation of the structural distances among PSNs in our sample. The MDS solution reveals a striking tripartite pattern of differentiation among the cysteine proteases, with protein structures exhibiting continuous and unilateral variation along three nearly orthogonal axes. A four-group hierarchical clustering solution (using Ward's method) on the underlying distance data is consonant with this pattern, yielding one cluster for each “spoke” of the tripartite structure (red, green, and blue points) and one cluster associated with the central “hub” (black points). The structure with the smallest median distance to all other structures (also the smallest maximum distance) is Aspain (orange point); notable reference structures within the central hub include papain and zingipain, which are in this sense among the most “typical” structures in the set. Oryzain characterizes the extreme end of the red spoke, which includes most of the proteins possessing a granulin domain (the remaining cases extending into the red sector of the central hub). The most extreme structure in the blue spoke is DCAP_2555 with other members including references SAG39 and Dionain 3. Lastly, the green extreme corresponds to DCAP_4793; while this spoke contains almost exclusively *D. capensis* proteins, Ervatamin B lies at the interface of this spoke group with the hub cluster.

Given that each “spoke” in the MDS solution represents a distinct mode of variation, it is natural to suspect that the corresponding differences are structurally well-localized. Insets (b)–(d) of Fig. 8 show that this is not the case. Each inset shows the minimum distance mapping between the most extreme PSN on one MDS spoke and the central PSN (Aspain), with vertices placed using a standard (Fruchterman–Reingold) layout algorithm. Black edges within each network correspond to vertex interactions that are consistent between both structures, while red and blue edges reflect respectively interactions that are found only at the spoke extreme or central PSN. If structural differences along each spoke were well-localized, then red and blue regions within each inset would be concentrated within a particular part of the network structure; instead, we see a large number of “clouds” of red and blue edges in each network, indicating that systematic differences are found at numerous locations within the protein structure. Fig. 8e provides a detailed view of one such comparison. As can be seen, concentrations of red and blue edges tend to be separated by vertex sets that are well-connected by common (black) edges, indicating that the spoke differences typically correspond to broad shifts in the neighborhoods of large numbers of adjoining chemical groups (e.g., as produced by wholesale rotation or translation of large secondary structure elements). Each spoke contains several such rearrangements, extending throughout the entire protein structure, rather than either a very large number of idiosyncratic local changes or the reorganization of one portion of the protein.

This PSN-based analysis complements our findings regarding specific local features by suggesting that global differences in the structure of interaction among chemical groups fall along a small number of axes, from which it is then straightforward to select candidates for subsequent expression and biophysical characterization. By turns, the analysis also helps identify proteases with more “conventional” structures (e.g., papain, Aspain) that may serve as points of comparison vis a vis those from other groups.

## 4 Conclusion

In summary, 44 cysteine proteases were identified directly from the genomic DNA of *D. capensis*, and sorted into clusters based on sequence homology to known plant cysteine proteases. Molecular modeling and network analysis indicate that these proteases have distinct structural properties suggesting potential diversity in functional characteristics (e.g., thermal stability, substrate affinity). These diverse properties make this class of proteins an attractive target for further characterization studies, with rich potential for biotechnology applications.

One particularly attractive potential application for these proteases is in mass spectrometry-based proteomics. In bottom-up or shotgun proteomics, the proteins under investigation are digested into fragments using one or more proteases, followed by LC–MS/MS analysis [73]. In the most commonly performed experiments, the digestion is performed using trypsin, which is chosen because its propensity for producing fragments containing at least one basic (positively charged) residue makes it convenient for use with collisional activated tandem MS [74]. However, for investigating complex protein mixtures, using a combination of proteases with different specificities improves proteome sequence coverage; this is particularly true for proteins that are present at low abundance [75]. On the other hand, electron transfer or electron capture dissociation methodology does not depend on the presence of basic residues in the individual peptides, allowing more flexibility in the choice of proteases for digestion [76]. The use of multiple proteases also enables better characterization of post-translational modifications [77]. Identification and characterization of new proteases from diverse sources, including carnivorous plants, adds to the repertoire of cleavage patterns that can be used in proteomics research.

Furthermore, the proteases from *D. capensis* are particularly attractive for mass spectrometry applications because they are optimized to function at pH 5. The optimal pH range of trypsin and other commonly used proteases, such as chymotrypsin and LysC, is neutral to mildly basic (7.8–8). This can be problematic for proteomic studies because at this pH, spontaneous deamidation of N and Q residues often occurs via formation of a succinimide intermediate [78], [79]. This type of modification during sample preparation for mass spectrometry is relatively common [80], [81], [82]. Worse, it is not randomly distributed but depends on the neighboring residues [83], leading to artifactual modifications that can provide misleading results about protein aging [84], [85] and the sites of N-linked glycosylation [86]. It has recently been shown that deamidation can be avoided by preparing the sample under slightly acidic conditions [87], a process potentially facilitated by the availability of *D. capensis* proteases. Disulfide scrambling can also be minimized by working at lower pH [88]. Future studies will include simulation of enzyme activity as a function of pH, as recently demonstrated for RNase A [89], as well as experimental characterization.

## Author contributions

C.T.B. performed the cluster analysis, molecular dynamics simulations, and network visualization and analysis. X.Z., J.A.F., and C.T.B. created the protein structure networks. R.W.M. chose the protein set, generated the Rosetta structures, and performed sequence comparisons and structure analysis. J.E.K., K.W.R., M.H.U., and S.T. performed sequence annotation and comparisons. R.W.M. and C.T.B. designed the study and wrote the manuscript.

## Appendix A Supplementary data

Supplementary material. Image 2

Image 2

Image 2

Image 2
