# Supplementary material for: Sequence comparison, molecular modeling, and network analysis predict structural diversity in cysteine proteases from the Cape sundew, Drosera capensis
Source: Comput Struct Biotechnol J. 2016 Jun 14;14:271–82. doi: 10.1016/j.csbj.2016.05.003 (PMC4949590; doi:10.1016/j.csbj.2016.05.003)
Supplement: Supplementary file 1 — Supplementary material. [file mmc1.pdf]

Sequence comparison, molecular modeling, and network analysis predict structural diversity in cysteine proteases from the Cape sundew, *Drosera capensis*: Supplementary Data

Carter T. Butts,[1,2,3*] Xuhong Zhang,[3] John E. Kelly,[4] Kyle W. Roskamp,[4] Megha H. Unhelkar,[4] J. Alfredo Freites,[4] Seemal Tahir,[4] and Rachel W. Martin[4,5*]

[1]*Department of Sociology, UC Irvine,*
[2]*Department of Statistics, UC Irvine*
[3]*Department of Electrical Engineering and Computer Science, UC Irvine,*
[4]*Department of Chemistry, UC Irvine*
[5]*Department of Molecular Biology & Biochemistry, UC Irvine*
Irvine, CA, 92697 USA
[*]*To whom correspondence should be addressed; E-mail: rwmartin@uci.edu, buttsc@uci.edu.*

Cysteine Protease Sequence Analysis

Multiple-sequence alignments for cysteine proteases from *D. capensis* and previously characterized plant cysteine proteases reveal diverse functionality. Annotated sequence alignments are shown for the DCAP cluster (Figure S1), the papain cluster, both catalytic domains (Figure S2) and (Figure S3), the vignain cluster, (Figure S4), the granulin domain cluster (Figure S5), the bromelain cluster (Figure S6) and the dionain cluster (Figure S7). The annotations highlight both specific amino acid properties and general sequence features. Hydrophobic residues are shown in green, positively charged residues in blue, negatively charged residues in red, and cysteines in yellow. Conserved Cys residues involved in structure-stabilizing disulfide bonds are indicated with yellow asterisks, while other residues conserved across all the sequences considered are indicated with solid circles. Residues conserved within the cluster but not shared with papain are indicated with open circles. The residues of the catalytic dyad are indicated with colored arrows, yellow for Cys and purple for His. The position of the sta-

bilizing Asn residue is indicated with a pink asterisk, although this residue is not conserved in all sequences. Strikethrough text indicates parts of the sequence that are expressed but removed during post-translational processing; for these proteins, this constitutes an N-terminal region comprised of the signal peptide and the pro-sequence. The presence and position of a signal sequence targeting the protein for secretion was predicted using SignalP, and is indicated in the figures by highlighting in light orange, with the predicted cut site indicated by underlining the residues on either side of the cleavage point. The position of the pro-sequences was predicted by sequence similarity to the reference sequences as well as comparison of the predicted structures to the crystal structure of the mature form of papain. The pro-seqeunces of many of the sequences studied here contain the ERFNIN motif ($EX_3RX_3FX_2NX_3I/VX_3N$) common to C1-family cysteine proteases. When present, this sequence is shown above the relevant residues in the alignments. The presence of localization tags, when present, is indicated by purple highlighting. Granulin domains, when present, are highlighted in blue.

Sequence consensus analysis for the sequences within each cluster defined in Figure S1, are mapped onto the structure of a representative member of the class in Figure S8. Percent conservation at each position is color coded (red = more conserved, white = intermediate values, blue = less conserved). These plots demonstrate that the degree of sequence conservation varies greatly among different clusters, i.e. the DCAP cluster has much less sequence conservation overall than the vignain cluster. In all the clusters, conserved residues are concentrated in the important secondary structure elements and near the active site cleft, whereas residues in loops and linkers away from the core of the protein are less likely to be conserved.

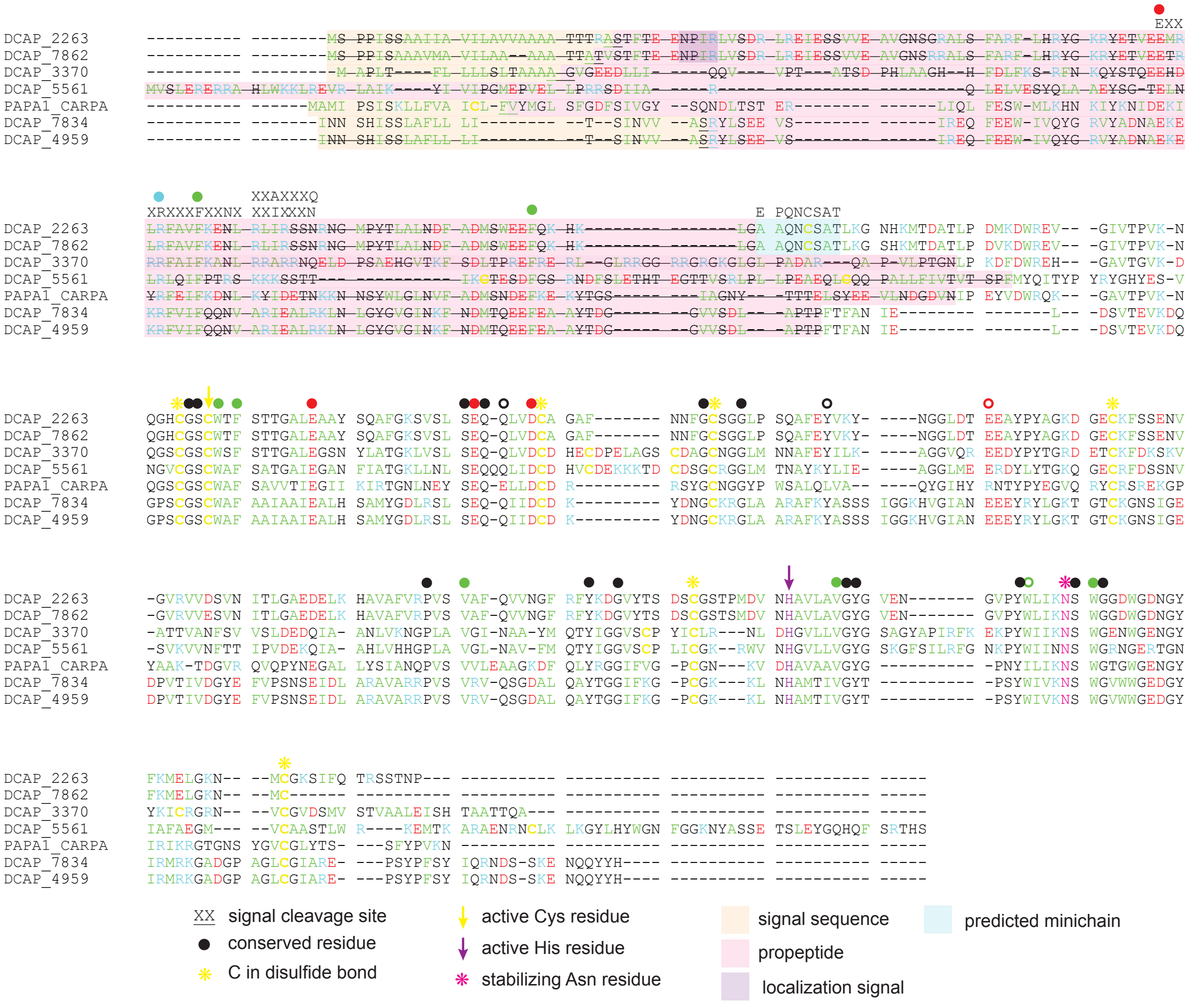

Figure S1: The DCAP cluster contains sequences that are more closely related to other *D. capensis* sequences than to any of the references. Several have insertions not found in other sequences, potentially indicating specific functionalities. DCAP_2263 and DCAP_7862 contain the localization tag NPIR in their N-terminal pro-domain regions, indicating targeting to the vacuole.

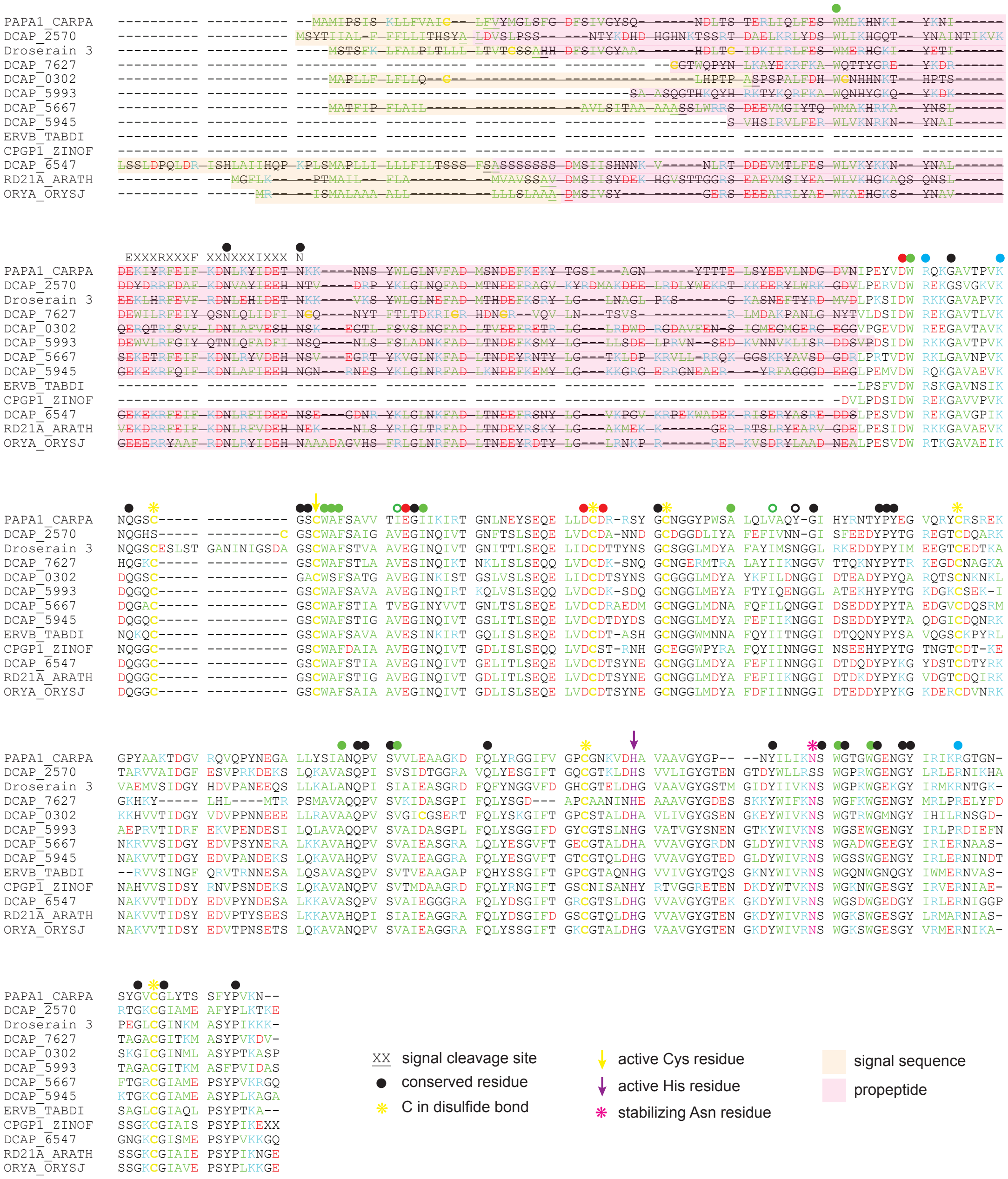

Figure S2: Many of the reference sequences belong to the papain cluster despite the diversity of their sources.. Several proteins in cluster also have C-terminal granulin domains, which are shown in Fig. SS3.

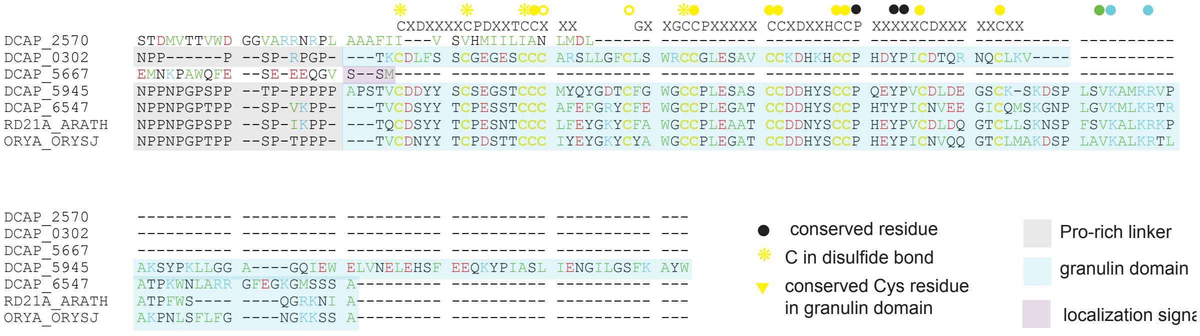

Figure S3: The papain cluster granulin domains contain several examples homologous to the reference proteins RD21_ARATH and ORYA_ORYSJ. Papain itself lacks a C-terminal granulin domain, so it is not included in the alignment. DCAP_2570 and DCAP_5667 are truncated, and therefore do not contain both disulfide bonds stabilizing the granulin domains. DCAP_ 5945 contains an extra C-terminal extension not found in the reference sequences. The conserved sequence region characterizing animal granulin domains is shown above the corresponding sequences for comparison. The plant granulin sequences have two distinguishing features; an additional conserved Cys residue is present immediately after the first conserved CC pair in the animal sequence, and a 6-residue insertion containing another conserved C is present between the first and second CC pairs.

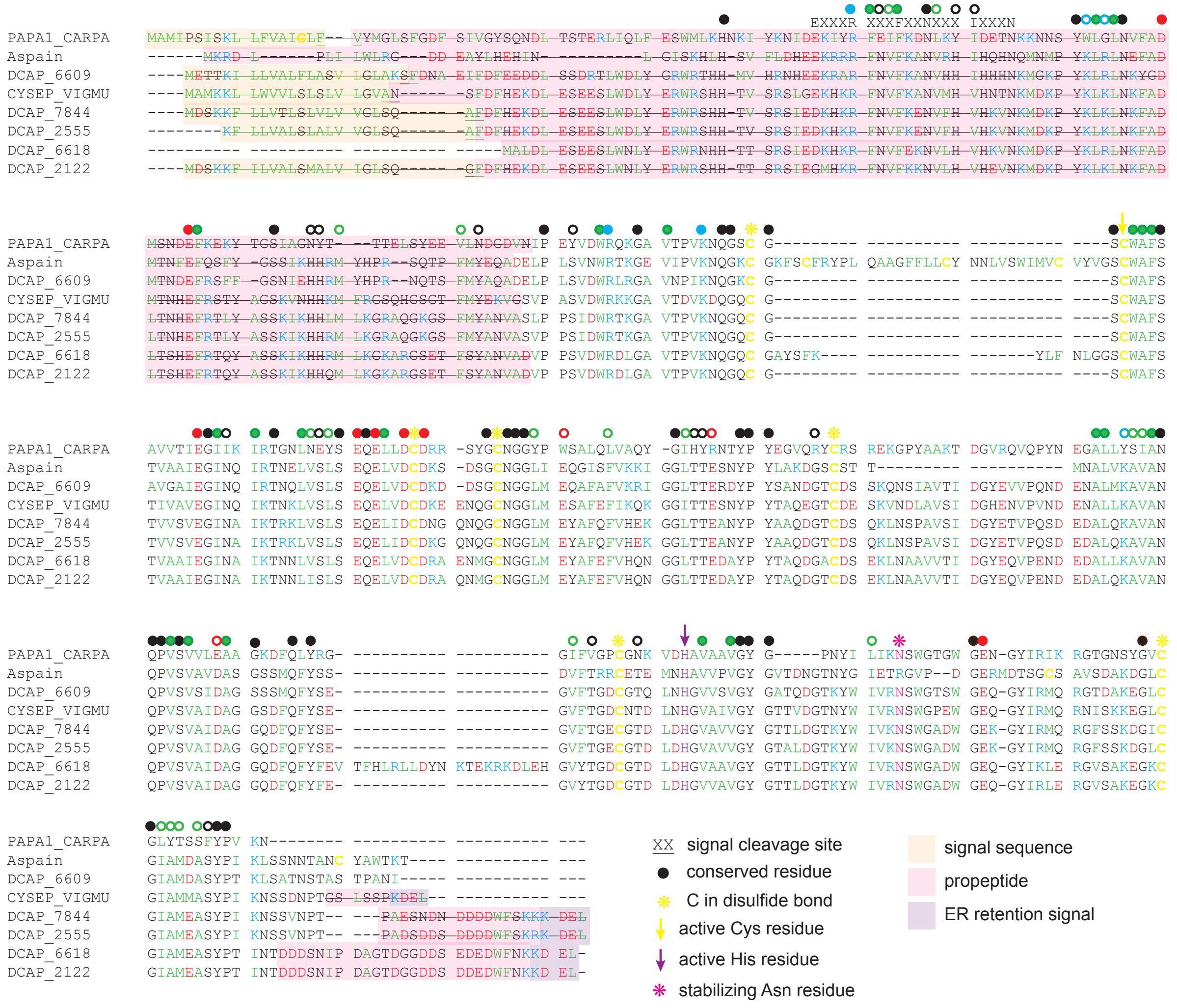

Figure S4: Many proteins in the vignain cluster, including vignain itself, are characterized by the localization tag KDEL at the C-terminus. This sequence element indicates that the protein is marked for retention in the endoplasmic reticulum.

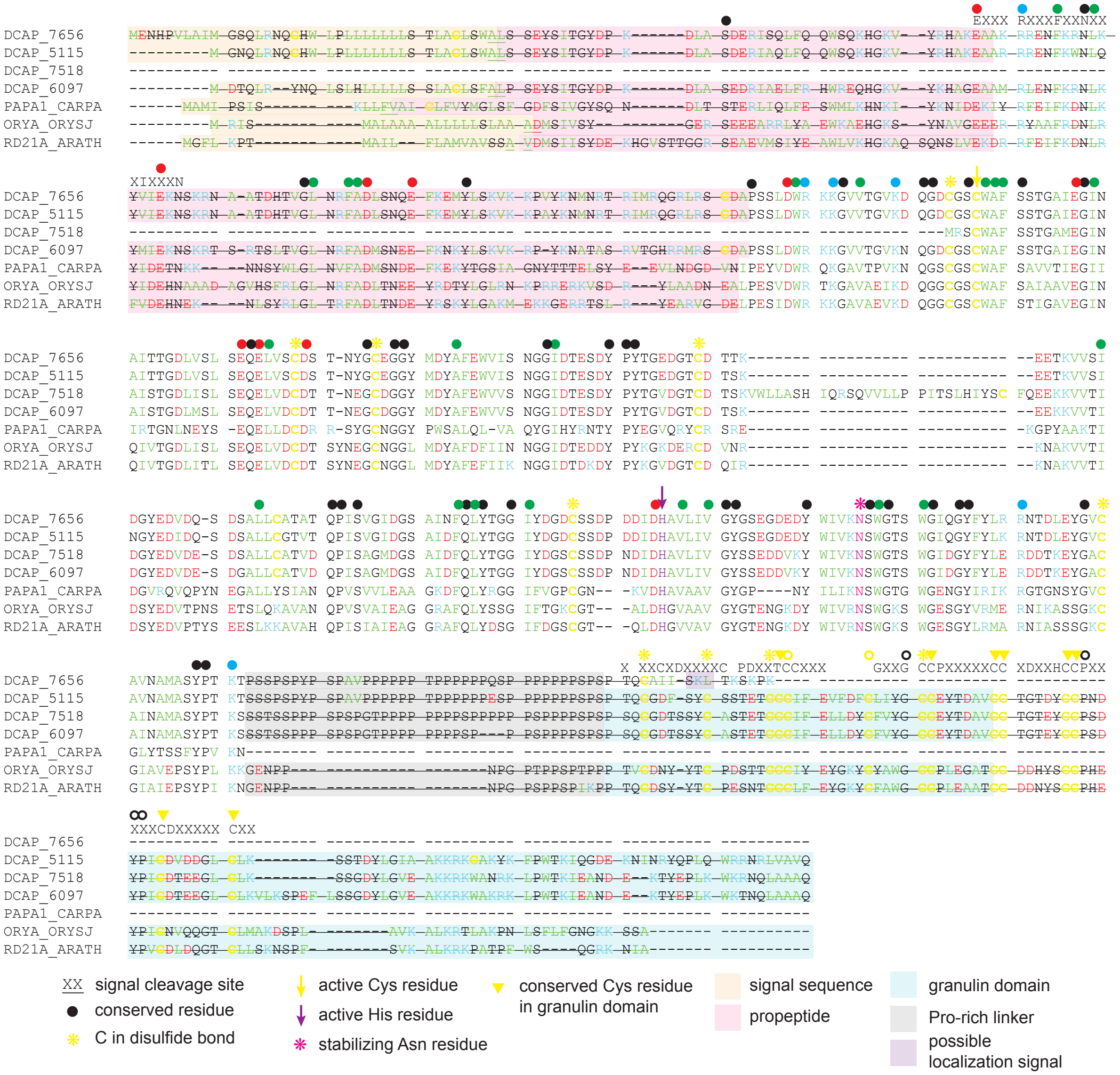

Figure S5: The granulin domain cluster contains proteins with C-terminal granulin domains. Although they are not closely related to any of the reference sequences, RD21_ARATH and ORYA_ORYSJ are shown in the alignment in order to compare sequence features among the granulin domains. As shown for the papain cluster granulin domains, the conserved sequence region characterizing animal granulin domains placed above the corresponding sequences. As in the papain case, there are two additional conserved Cs and a 6-residue insertion between the first and second CC pairs. In these sequence, a deletion of one residue relative to the animal sequence also occurs between the first and second conserved Cys residues in the granulin domain. DCAP_7656 is missing most of the granulin domain, and instead contains the localization tag SKL near the C-terminus, marking it for transport to the peroxisome.

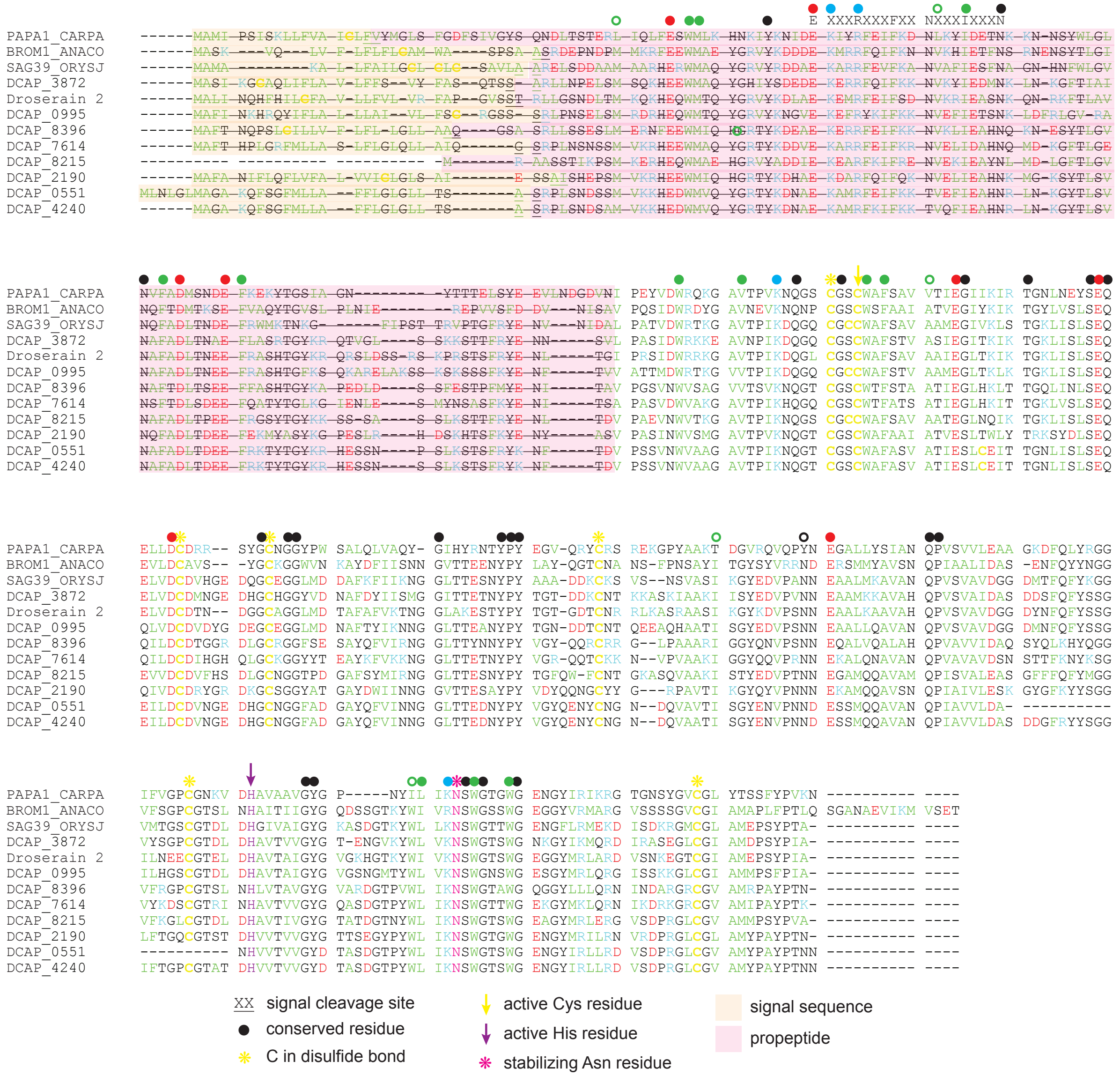

Figure S6: The bromelain cluster is characterized by strong sequence identity with pineapple fruit bromelain.

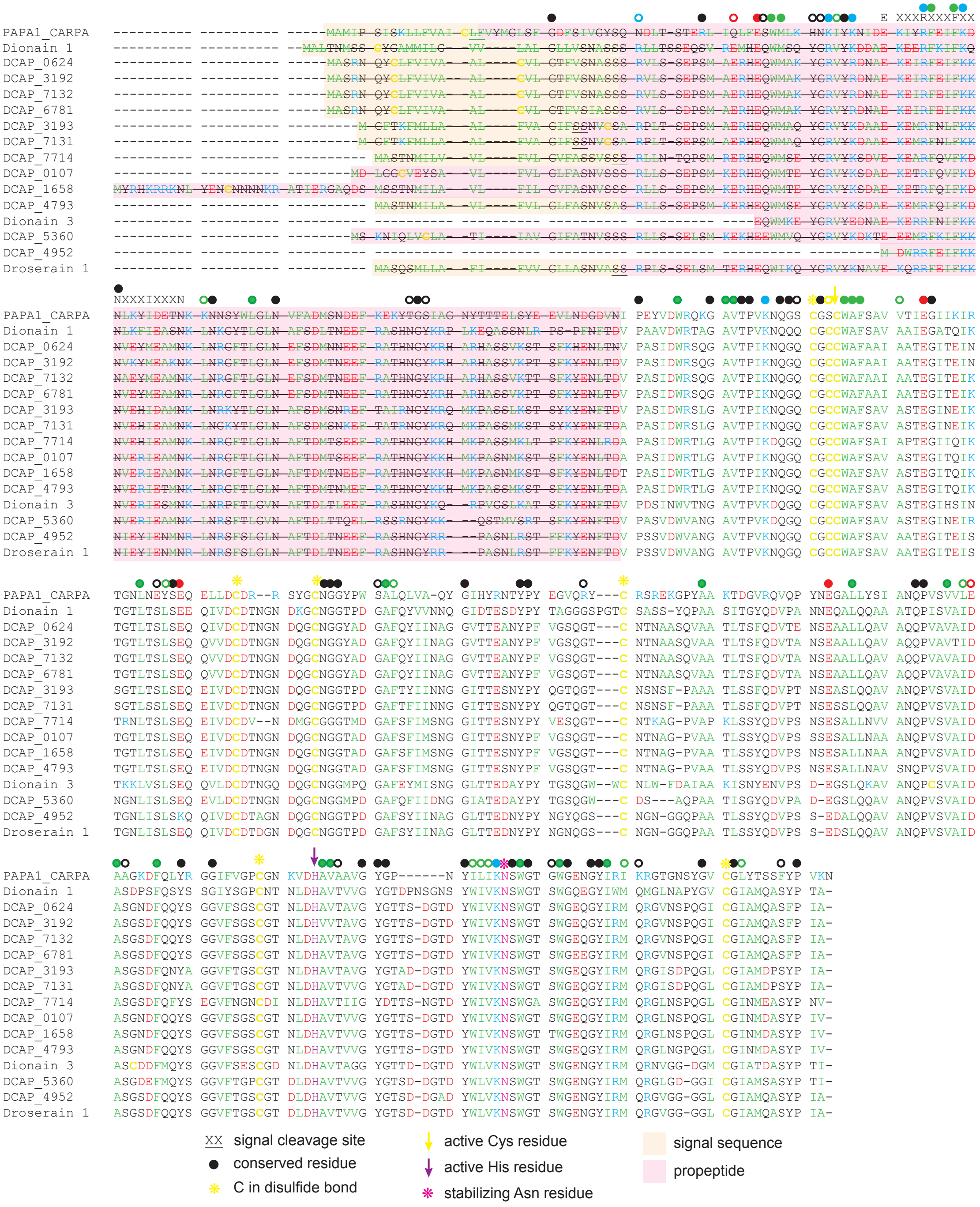

Figure S7: The dionain cluster contains many cysteine proteases that appear to be specific to Caryophylalles carnivorous plants; this cluster contains the dionains from *D. muscipula* as well as several proteins from *D. capensis*, but none of the reference sequences from other sources.

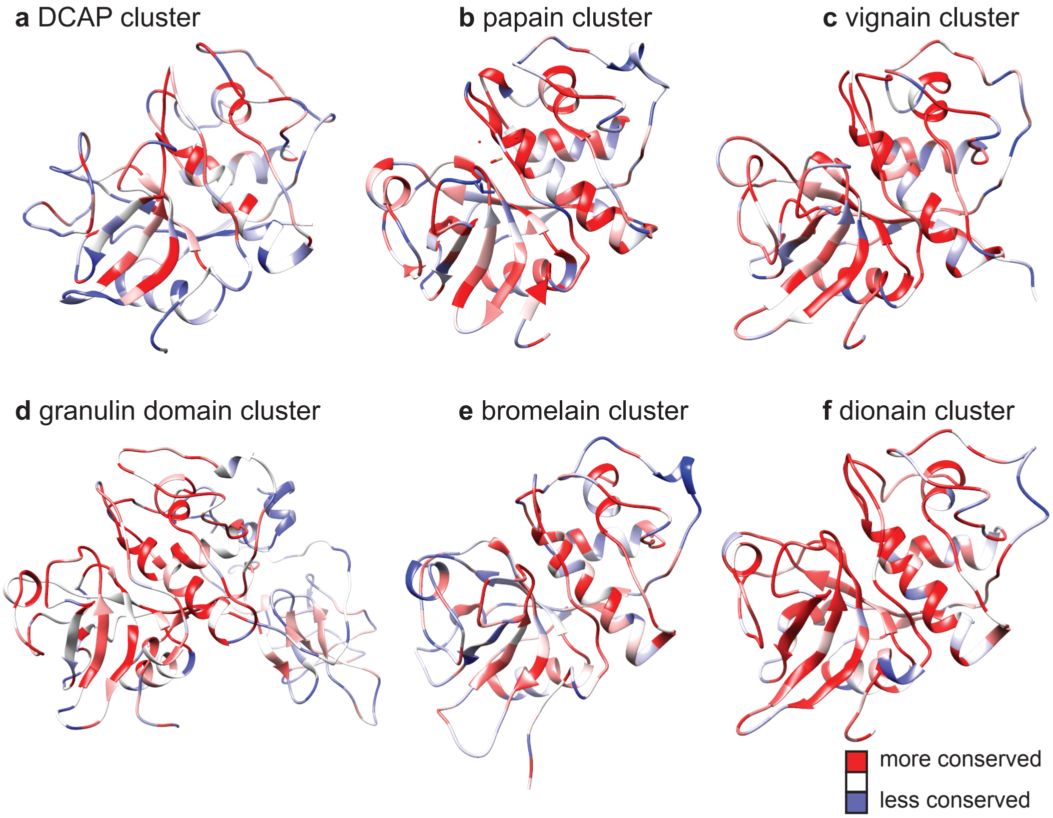

Figure S8: The percent conservation of each residue in the consensus sequence for each cluster is shown mapped onto a representative member of the cluster. The color scale ranges from red (more conserved) to blue (less conserved). a. DCAP cluster (DCAP_2263) b. papain cluster (papain) c. vignain cluster ((DCAP_2122) d. granulin domain cluster (DCAP_5115) e. bromelain cluster (droserain 2) and f. dionain cluster (DCAP_0624).

Supplementary Table 1: Taxonomy Table

protein name	protein abbreviation	organism	order	family
papain	PAPA1_CARPA	Papaya- *Carica papaya*	Brassicales	Caricaceae
zingipain 1	CPGP1_ZINOF	Ginger- *Zingiber officianale*	Zingiberales	Zingiberaceae
fruit bromelain	BROM1_ANACO	Pineapple- *Ananas cosmosus*	Poales	Bromeliaceae
oryzain	ORYA_ORYSJ	Rice- *Oryza sativa*	Poales	Poaceae
SAG39	SAG39_ORYSJ	Rice- *Oryza sativa*	Poales	Poaceae
RD21	RD21A_ARATH	Arabidopsis- *Arabidopsis thaliana*	Brassicales	Brassicaceae
ervatamin B	ERVB_TABDI	Crepe jasmine- *Tabernaemontana divaricata*	Gentianales	Apocynaceae
vignain	CYSEP_VIGMU	Mung bean- *Vigna mungo*	Fabales	Fabaceae
droserain 1	droserain 1	Venus flytrap- *Dionaea muscipula*	Caryophylalles	Droseraceae

0.1. Reference sequence sources

Reference sequences used in this work come from a variety of different plant sources representing diverse families. The reference sequences and their sources are tabulated in Table S1.

Three-dimensional structure prediction

Three-dimensional structures were predicted for all the proteases described in this study using a three-stage process:

1. Where known PDB structures were available, these were used as initial estimates. For cases in which no structure is available, initial estimates of protein structure were obtained via the Robetta server [1] using the full sequence as input.
2. Initial structures obtained in step (1) were then processed as follows. For structures obtained from x-ray crystallography, all heteroatoms were removed and the protein was protonated using REDUCE [2]. These and Rosetta-generated structures were then modified by removal of all signal sequences, pro-sequences, or other residues not found in the mature protein. Disulfide bonds in the remaining protein structure were then identified by a combination of structural proximity and homology to a common reference (RD21A_ARATH), and the structure modified accordingly. Finally, the Cys and His residues in the active site were respectively deprotonated and fully protonated, resulting in the matured but unequilibrated structure.
3. The matured structure in (2) was then equilibrated in explicit solvent using NAMD [3] using the CHARMM22 forcefield [4] with the CMAP correction [5] and the TIP3P water model [6]. Using VMD [7], each structure was solvated within a box of minimum 10 Angstrom margin on all sides, and Na^{+} or Cl^{-} ions were added as necessary to neutralize the charge of the resulting structure. The resulting ensemble was minimized under periodic

boundary conditions at 293K and 1 atm for 10000 iterations (with temperature controlled via Langevin dynamics with a damping coefficient of 1, and pressure controlled via the combined Nosé-Hoover/Langevin piston method [8, 9]), and then simulated for beween 0.5-2ps to allow for Langevin piston adjustment. The box size was then recalibrated, and the ensemble was then simulated for a further 500ps under identical conditions. The final protein conformation was retained for subsequent analysis.

In the case of proteins with granulin domains, structures were produced for both domain-attached and domain-removed cases. The PDB files corresponding to the predicted structures (listed in Supplementary Tables 1 and 2) are available for download in the Supplementary Material.

Supplementary Table 2: Cysteine protease Rosetta structure PDB files available for download.

protein	organism	sequence elements included	file name
Aspain	*D. capensis*	pro-seq., active region	Aspain_full_m1.pdb
DCAP_3370	*D. capensis*	signal, pro-seq., active region	DCAP_3370_full_m1.pdb
DCAP_5561	*D. capensis*	pro-seq. (N), active region	DCAP_5561_full_m1.pdb
DCAP_2263	*D. capensis*	signal, pro-seq. (N), active region	DCAP_2263_full_m1.pdb
DCAP_7862	*D. capensis*	signal, pro-seq. (N), active region	DCAP_7862_full_m1.pdb
DCAP_7834	*D. capensis*	signal, pro-seq. (N), active region	DCAP_7834_full_m1.pdb
DCAP_4959	*D. capensis*	signal, pro-seq. (N), active region	DCAP_4959_full_m1.pdb
DCAP_0302	*D. capensis*	signal, pro-seq. (N), active region, P-rich linker, granulin	DCAP_0302_full_m1.pdb
DCAP_6547	*D. capensis*	signal, pro-seq. (N), active region, P-rich linker, granulin	DCAP_6547_full_m1.pdb
DCAP_5945	*D. capensis*	pro-seq. (N), active region, P-rich linker, granulin	DCAP_5945_full_m1.pdb
DCAP_5667	*D. capensis*	signal, pro-seq. (N), active region	DCAP_5667_full_m1.pdb
DCAP_5993	*D. capensis*	pro-seq. (N), active region	DCAP_5993_full_m1.pdb
DCAP_7627	*D. capensis*	pro-seq. (N), active region	DCAP_7627_full_m1.pdb
DCAP_2570	*D. capensis*	signal, pro-seq. (N), active region	DCAP_2570_full_m1.pdb
DCAP_2555	*D. capensis*	signal, pro-seq.(N), active region, pro-seq (C)	DCAP_2555_full_m1.pdb
DCAP_7844	*D. capensis*	signal, pro-seq.(N), active region, pro-seq (C)	DCAP_7844_full_m1.pdb
DCAP_2122	*D. capensis*	signal, pro-seq.(N), active region, pro-seq (C)	DCAP_2122_full_m1.pdb
DCAP_6618	*D. capensis*	pro-seq.(N), active region, pro-seq (C)	DCAP_6618_full_m1.pdb
DCAP_6609	*D. capensis*	signal, pro-seq., active region	DCAP_6609_full_m1.pdb
DCAP_7656	*D. capensis*	signal, pro-seq.(N), active region, P-rich linker, granulin	DCAP_7656_full_m1.pdb
DCAP_5115	*D. capensis*	signal, pro-seq.(N), active region, P-rich linker, granulin	DCAP_5115_full_m1.pdb
DCAP_7518	*D. capensis*	active region, P-rich linker, granulin	DCAP_7518_full_m1.pdb
DCAP_6097	*D. capensis*	signal, pro-seq.(N), active region, P-rich linker, granulin	DCAP_6097_full_m1.pdb
DCAP_3872	*D. capensis*	signal, pro-seq. (N), active region	DCAP_3872_full_m1.pdb
DCAP_8215	*D. capensis*	pro-seq. (N), active region	DCAP_8215_full_m1.pdb
DCAP_0995	*D. capensis*	signal, pro-seq. (N), active region	DCAP_0995_full_m1.pdb
DCAP_0551	*D. capensis*	signal, pro-seq. (N), active region	DCAP_0551_full_m1.pdb
DCAP_4240	*D. capensis*	signal, pro-seq. (N), active region	DCAP_4240_full_m1.pdb
DCAP_2190	*D. capensis*	signal, pro-seq. (N), active region	DCAP_2190_full_m1.pdb
DCAP_7614	*D. capensis*	signal, pro-seq. (N), active region	DCAP_7614_full_m1.pdb
DCAP_8396	*D. capensis*	signal, pro-seq. (N), active region	DCAP_8396_full_m1.pdb
DCAP_5360	*D. capensis*	pro-seq. (N), active region	DCAP_5360_full_m1.pdb
DCAP_4952	*D. capensis*	pro-seq. (N), active region	DCAP_4952_full_m1.pdb
DCAP_3193	*D. capensis*	signal, pro-seq. (N), active region	DCAP_3193_full_m1.pdb
DCAP_7131	*D. capensis*	signal, pro-seq. (N), active region	DCAP_7131_full_m1.pdb
DCAP_1658	*D. capensis*	pro-seq. (N), active region	DCAP_1658_full_m1.pdb
DCAP_4793	*D. capensis*	signal, pro-seq. (N), active region	DCAP_4793_full_m1.pdb
DCAP_0107	*D. capensis*	pro-seq. (N), active region	DCAP_0107_full_m1.pdb
DCAP_7714	*D. capensis*	signal, pro-seq. (N), active region	DCAP_7714_full_m1.pdb
DCAP_7132	*D. capensis*	signal, pro-seq. (N), active region	DCAP_7132_full_m1.pdb
DCAP_6781	*D. capensis*	signal, pro-seq. (N), active region	DCAP_6781_full_m1.pdb
DCAP_3192	*D. capensis*	signal, pro-seq. (N), active region	DCAP_3192_full_m1.pdb
DCAP_0624	*D. capensis*	signal, pro-seq. (N), active region	DCAP_0624_full_m1.pdb
Dionain 1	*D. muscipula*	signal, pro-seq. (N), active region	Dionain1_full_m1.pdb
Dionain 3	*D. muscipula*	pro-seq. (N), active region	Dionain3_full_m1.pdb
Droserain 1	*D. capensis*	signal, pro-seq. (N), active region	Droserain1_full_m1.pdb
Droserain 2	*D. capensis*	signal, pro-seq. (N), active region	Droserain2_full_m1.pdb
Droserain 3	*D. capensis*	signal, pro-seq. (N), active region	Droserain3_full_m1.pdb
Ervatamin B	*T. divaricata*	active region	ERVB_TABDI_full_m1.pdb
Fruit bromelain	*A comosus*	signal, pro-seq. (N), active region	BROM1_ANACO_full_m1.pdb
Oryzain	*O. sativa*	signal, pro-seq. (N), active region, P-rich linker, granulin	ORYA_ORYSJ_full_m1.pdb
Papain	*C. papaya*	signal, pro-seq. (N), active region	PAPA1_CARPA.pdb
RD21	*A. thaliana*	signal, pro-seq. (N), active region, P-rich linker, granulin	RD21A_ARATH_full_m1.pdb
SAG39	*O. sativa*	signal, pro-seq. (N), active region	SAG39_ORYSJ_full_m1.pdb
Vignain	*V. mungo*	signal, pro-seq.(N), active region, pro-seq (C)	CYSEP_VIGMU_full_m1.pdb
Zingipain 1	*Z. officinale*	active region	CPGP1_ZINOF_full_m1.pdb

Supplementary Table 3: Equilibrated Cysteine protease Rosetta structure PDB files available for download.

protein	organism	sequence elements included	file name
Aspain	*D. capensis*	active region	Aspain_mature_m1.pdb
DCAP_3370	*D. capensis*	active region	DCAP_3370_mature_m1.pdb
DCAP_5561	*D. capensis*	active region	DCAP_5561_mature_m1.pdb
DCAP_2263	*D. capensis*	active region	DCAP_2263_mature_m1.pdb
DCAP_7862	*D. capensis*	active region	DCAP_7862_mature_m1.pdb
DCAP_7834	*D. capensis*	active region	DCAP_7834_mature_m1.pdb
DCAP_4959	*D. capensis*	active region	DCAP_4959_mature_m1.pdb
DCAP_0302	*D. capensis*	active region, P-rich linker, granulin	DCAP_0302_mature_m1.pdb
DCAP_0302	*D. capensis*	active region	DCAP_0302_nogranulin_mature_m1.pdb
DCAP_6547	*D. capensis*	active region, P-rich linker, granulin	DCAP_6547_mature_m1.pdb
DCAP_6547	*D. capensis*	active region	DCAP_6547_nogranulin_mature_m1.pdb
DCAP_5945	*D. capensis*	active region, P-rich linker, granulin	DCAP_5945_mature_m1.pdb
DCAP_5945	*D. capensis*	active region	DCAP_5945_nogranulin_mature_m1.pdb
DCAP_5667	*D. capensis*	active region	DCAP_5667_mature_m1.pdb
DCAP_5993	*D. capensis*	active region	DCAP_5993_mature_m1.pdb
DCAP_7627	*D. capensis*	active region	DCAP_7627_mature_m1.pdb
DCAP_2570	*D. capensis*	active region	DCAP_2570_mature_m1.pdb
DCAP_2555	*D. capensis*	active region	DCAP_2555_mature_m1.pdb
DCAP_7844	*D. capensis*	active region	DCAP_7844_mature_m1.pdb
DCAP_2122	*D. capensis*	active region	DCAP_2122_mature_m1.pdb
DCAP_6618	*D. capensis*	active region	DCAP_6618_mature_m1.pdb
DCAP_6609	*D. capensis*	active region	DCAP_6609_mature_m1.pdb
DCAP_7656	*D. capensis*	active region, P-rich linker	DCAP_7656_mature_m1.pdb
DCAP_5115	*D. capensis*	active region, P-rich linker, granulin	DCAP_5115_mature_m1.pdb
DCAP_5115	*D. capensis*	active region	DCAP_5115_nogranulin_mature_m1.pdb
DCAP_7518	*D. capensis*	active region, P-rich linker, granulin	DCAP_7518_mature_m1.pdb
DCAP_7518	*D. capensis*	active region	DCAP_7518_nogranulin_mature_m1.pdb
DCAP_6097	*D. capensis*	active region, P-rich linker, granulin	DCAP_6097_mature_m1.pdb
DCAP_6097	*D. capensis*	active region	DCAP_6097_nogranulin_mature_m1.pdb
DCAP_3872	*D. capensis*	active region	DCAP_3872_mature_m1.pdb
DCAP_8215	*D. capensis*	active region	DCAP_8215_mature_m1.pdb
DCAP_0995	*D. capensis*	active region	DCAP_0995_mature_m1.pdb
DCAP_0551	*D. capensis*	active region	DCAP_0551_mature_m1.pdb
DCAP_4240	*D. capensis*	active region	DCAP_4240_mature_m1.pdb
DCAP_2190	*D. capensis*	active region	DCAP_2190_mature_m1.pdb
DCAP_7614	*D. capensis*	active region	DCAP_7614_mature_m1.pdb
DCAP_8396	*D. capensis*	active region	DCAP_8396_mature_m1.pdb
DCAP_5360	*D. capensis*	active region	DCAP_5360_mature_m1.pdb
DCAP_4952	*D. capensis*	active region	DCAP_4952_mature_m1.pdb
DCAP_3193	*D. capensis*	active region	DCAP_3193_mature_m1.pdb
DCAP_7131	*D. capensis*	active region	DCAP_7131_mature_m1.pdb
DCAP_1658	*D. capensis*	active region	DCAP_1658_mature_m1.pdb
DCAP_4793	*D. capensis*	active region	DCAP_4793_mature_m1.pdb
DCAP_0107	*D. capensis*	active region	DCAP_0107_mature_m1.pdb
DCAP_7714	*D. capensis*	active region	DCAP_7714_mature_m1.pdb
DCAP_7132	*D. capensis*	active region	DCAP_7132_mature_m1.pdb
DCAP_6781	*D. capensis*	active region	DCAP_6781_mature_m1.pdb
DCAP_3192	*D. capensis*	active region	DCAP_3192_mature_m1.pdb
DCAP_0624	*D. capensis*	active region	DCAP_0624_mature_m1.pdb
Dionain 1	*D. muscipula*	active region	Dionain1_mature_m1.pdb
Dionain 3	*D. muscipula*	active region	Dionain3_mature_m1.pdb
Droserain 1	*D. capensis*	active region	Droserain1_mature_m1.pdb
Droserain 2	*D. capensis*	active region	Droserain2_mature_m1.pdb
Droserain 3	*D. capensis*	active region	Droserain3_mature_m1.pdb
Ervatamin B	*T. divaricata*	active region	ERVB_TABDI_mature_m1.pdb
Fruit bromelain	*A comosus*	active region	BROM1_ANACO_mature_m1.pdb

Supplementary Table 3 con't: Equilibrated Cysteine protease Rosetta structure PDB files available for download.

protein	organism	sequence elements included	file name
Oryzain	*O. sativa*	active region, P-rich linker, granulin	ORYA_ORYSJ_mature_m1.pdb
Oryzain	*O. sativa*	active region	ORYA_ORYSJ_nogranulin_mature_m1.pdb
Papain	*C. papaya*	active region	PAPA1_CARPA_mature_m1.pdb
RD21	*A. thaliana*	active region, P-rich linker, granulin	RD21A_ARATH_mature_m1.pdb
SAG39	*O. sativa*	active region	SAG39_ORYSJ_mature_m1.pdb
Vignain	*V. mungo*	active region	CYSEP_VIGMU_mature_m1.pdb
Zingipain 1	*Z. officinale*	active region	CPGP1_ZINOF_mature_m1.pdb

Protein Structure Network Analysis

Protein structure networks (PSNs) were created for each protein structure via custom scripts using VMD [7] and `statnet` [10] tools. The resulting PSNs were stored as a list of network objects [11], and can be obtained from the downloadable R file `protein_networks.RData`. Network objects are included for both the initial (minimized, pre-equilibration) and equilibrated structures, as distinguished by the respective suffices `.eq.0` and `.eq.1` in the "name" network attribute of each object.

Structural distances were calculated for all pairs of equilibrated structures using a version of the Butts and Carley [12] method. Unlabeled distances with an underlying Hamming metric were employed, corresponding to the minimum number of edge changes needed to take a member of the first graph's isomorphism class into a member of the second's. Distance computation for each graph pair was performed as follows. First, isolate addition was employed if necessary to obtain graphs of the same order. Second, hill-climbing on the space of all single pair permutations was used to find an initial local minimum distance. Third, simulated annealing was employed (initial temperature 8, 2e7 iterations, cooling factor 1-2e-7, 10 restarts) to obtain an additional estimate, which was further refined to a local minimum using hill-climbing. Finally, the minimum of the two estimates was employed for purposes of analysis. Due to the relatively large size of the graphs involved, a custom sparse-graph implementation of these algorithms was employed.

Analysis of the resulting distance matrix was performed as follows. First, all distances were normalized by vertex count (bearing in mind the isolate addition criterion above), yielding a metric in terms of edge changes per vertex. Next, the resulting distance matrix was analyzed using classical metric MDS (using the `cmdscale` function with R) and the tripartite structure verified using hierarchical clustering (using `hclust` with method `ward.D2`). Pairwise network comparisons were performed by selecting the central and extreme PSNs (as described in the text) and identifying the Hamming-minimizing cross-graph labeling using the minimization procedure described above. The remapped networks were then visualized using the `gplot` function within the `sna` library [13].

[1] D. Kim, D. Chivian, D. Baker, Protein structure prediction and analysis using the Robetta server, Nucleic Acids Research 32 (2004) W526–31.

[2] J. M. Word, S. C. Lovell, J. S. Richardson, D. C. Richardson, Asparagine

and glutamine: using hydrogen atom contacts in the choice of side-chain amide orientation, Journal of Molecular Biology 285 (1999) 1735–1747.

[3] J. C. Phillips, R. Braun, W. Wang, J. Gumbart, E. Tajkhorshid, E. Villa, C. Chipot, R. D. Skeel, L. Kal, K. Schulten, Scalable molecular dynamics with NAMD, Journal of Computational Chemistry 26 (2005) 1781–1802.

[4] A. D. MacKerell, D. Bashford, M. Bellott, R. L. Dunbrack, J. D. Evanseck, M. J. Field, S. Fischer, J. Gao, H. Guo, S. Ha, D. Joseph-McCarthy, L. Kuchnir, K. Kuczera, F. T. K. Lau, C. Mattos, S. Michnick, T. Ngo, D. T. Nguyen, B. Prodhom, W. E. Reiher, B. Roux, M. Schlenkrich, J. C. Smith, R. Stote, J. Straub, M. Watanabe, J. Wirkiewicz-Kuczera, D. Yin, M. Karplus, All-Atom Empirical Potential for Molecular Modeling and Dynamics Studies of Proteins, The Journal of Physical Chemistry B 102 (1998) 3586–3616.

[5] A. D. Mackerell, M. Feig, C. L. Brooks, Extending the treatment of backbone energetics in protein force fields: limitations of gas-phase quantum mechanics in reproducing protein conformational distributions in molecular dynamics simulations, Journal of Computational Chemistry 25 (2004) 1400–1415.

[6] W. L. Jorgensen, J. Chandrasekhar, J. D. Madura, R. W. Impey, M. L. Klein, Comparison of simple potential functions for simulating liquid water, The Journal of chemical physics 79 (1983) 926–935.

[7] W. Humphrey, A. Dalke, K. Schulten, VMD: visual molecular dynamics, Journal of Molecular Graphics 14 (1996) 33–38, 27–28.

[8] G. J. Martyna, D. J. Tobias, M. L. Klein, Constant pressure molecular dynamics algorithms, The Journal of Chemical Physics 101 (1994) 4177–4189.

[9] S. E. Feller, Y. Zhang, R. W. Pastor, B. R. Brooks, Constant pressure molecular dynamics simulation: The Langevin piston method, The Journal of Chemical Physics 103 (1995) 4613–4621.

[10] M. S. Handcock, D. R. Hunter, C. T. Butts, S. M. Goodreau, M. Morris, statnet: Software tools for the representation, visualization, analysis and simulation of network data, Journal of Statistical Software 24 (2008) 1–11.

[11] N. C. Benson, V. Daggett, A chemical group graph representation for efficient high-throughput analysis of atomistic protein simulations, Journal of Bioinformatics and Computational Biology 10 (2012) 1250008.

[12] C. T. Butts, K. M. Carley, Some simple algorithms for structural comparison, Computational and Mathematical Organization Theory 11 (2005) 291–305.

[13] C. T. Butts, Social network analysis with sna, Journal of Statistical Software 24 (2008).
